# Opinion: Gavage Administration of MXene as a Route‐Specific Alternative to Intravenous Injection into the Bloodstream of Laboratory Animals for Reducing Systemic Nanotoxicity Risks in Immunosuppression and Post‐Transplantation Models with Bile Acid Modification

**DOI:** 10.1002/adhm.71055

**Published:** 2026-03-18

**Authors:** Alireza Rafieerad, M. Akif Rahman, Ahmad Amiri

**Affiliations:** ^1^ Institute for Molecular Biosciences Johann Wolfgang Goethe Universität Frankfurt am Main Germany; ^2^ Canada‐Italy Tissue Engineering Laboratory Institute of Cardiovascular Sciences Department of Physiology and Pathophysiology St. Boniface Hospital Research Centre Rady Faculty of Health Sciences University of Manitoba Winnipeg Canada; ^3^ Institute for Biology and Biotechnology of Plants University of Münster Münster Germany; ^4^ Department of Mechanical Engineering The University of Tulsa Tulsa Oklahoma USA; ^5^ Russell School of Chemical Engineering University of Tulsa Tulsa Oklahoma USA

**Keywords:** DFT calculation, gavage delivery of MXene, in vivo immunosuppression strategy, innovative bile‐acid surface modifications, intravenous injection alternative, reduced systemic nanotoxicity risks

## Abstract

Intravenous injection of engineered material dispersions into the bloodstream of laboratory animals has been a popular method for in vivo immunosuppression. It is also one of the primary approaches for delivering immunosuppressive and anti‐inflammatory drugs within the early stages of donor cell/organ transplantation. Due to their systemic interaction with blood circulation and immune cells, the administration is reported to act effectively in suppressing host immune responses. Recent studies on immunomodulatory nanomaterials, including MXenes, have reported their potential for immunosuppression and post‐transplantation applications. In particular, MXene nanosheets and quantum dots of specific composition/form possess immunosuppressive properties that prevent stem/progenitor cell and cardiac transplant rejection, including the treatment of allograft vasculopathy, an accelerated form of atherosclerosis in organ recipients. Despite these promising in vivo findings, concerns regarding long‐term blood nanotoxicity or particle accumulation in circulatory organs, such as the heart, liver, kidneys, and lungs, as well as the associated risks of metabolic changes and other potential adverse biological pathways, remain challenging for clinical applications. This opinion perspective proposes the rational concept of gavage administration of bile acid‐modified MXenes, which may alleviate these risks while retaining their intrinsic immunosuppressive roles. It also presents novel density functional theory calculations for cholic acid surface modification to enhance MXenes’ immune‐suppressive properties and synergistically interact with gut beneficial microbiomes for smart interaction, bringing this nanobiotechnology closer to real‐world scenarios.

## Graphical abstract

Recent studies reported immunosuppressive properties of specific MXene nanomaterials. Their intravenous injection into the bloodstream of laboratory animals has been a common delivery method to suppress systemic inflammation and prevent transplant rejection. Despite the obtained promising results as well as this method's relevance in the delivery of drugs at early stages post‐transplantation, concerns regarding the long‐term blood nanotoxicity or particle accumulation in circulatory organs, including the heart, liver, kidney, or lungs, and the associated risks to metabolic changes or other potential adverse effects on biological pathways, remain challenging for clinical applications. This opinion perspective proposes the idea of gavage administration of MXenes, which may alleviate these risks, while retaining their immunosuppressive impact to interact with immune cells. It also offers novel bile‐acid surface modification strategies to enhance MXenes' immunosuppressive properties and synergistically interact with gut beneficial microbiomes for smart dual functionalities, bringing this nanobiotechnology closer to real‐world scenarios.

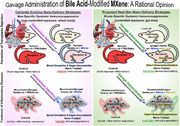

## 1 Introduction

Inflammation, a natural mechanism of the body's immune system in response to injuries and pathogens, can play a dual role, being both beneficial in healing and detrimental in progressing the chronic diseases. It can also result from the effects of host immune responses on donor cells, and organ transplantation is one of the long‐standing challenges in translational medical technology [1, 2]. To alleviate local or systemic inflammation and avoid the rejection of transplanted cells/organs, immunosuppressant therapies are required [3, 4]. This includes edible drugs or intravenous injection of immunosuppressive medications into the bloodstream at the early stages of transplantation, followed by oral administration of chemical drugs, which is vital for the rest of the recipients’ lives [5]. With progress in medical technologies, the outcomes of hybrid immune therapies have been proven effective in suppressing the inflammation and weakening the host immune system to prevent donor transplant rejection [6, 7]. These technologies have proven effective in inflammation suppression and reducing the associated risks of early rejection of transplanted organs, as well as the survival of allogenic stem cells in pre‐clinical trials. However, several side effects and adverse impacts of immunosuppressive drugs accompany the obtained advancements in the prevention of transplant rejection and treatment of autoimmune diseases [8, 9]. Thus, over recent years, extensive attention has been given to nanomedicine for designing and developing alternative strategies based on the application of low‐dimensional bioactive materials with smart immunosuppressive properties [10, 11]. The goal has been to apply nanotechnology to mitigate the adverse effects of immunosuppressive drugs or enhance their efficacy through combined therapies.

More recently, novel bio‐applications of transition‐metal carbide/nitride MXenes, which are the latest, most tunable, and largest discovered family of carbon‐based nanomaterials, have been introduced for immunomodulation or inducing anti‐inflammatory/viral mechanisms [12, 13, 14, 15, 16, 17, 18, 19, 20, 21, 22, 23]. MXene has a chemical formula of M_n+1_X_n_T_x_ (where n = 1–4, “M” stands for one or multiple early transition metal elements, “X” is C/N, and T_x_ signifies oxygen/fluorine/chlorine‐based surface functional terminations) [24, 25, 26]. In 2019, intending to suppress inflammation and smart immunoengineering, a pilot work study reported the first immunosuppression application of MXene to enhance the survival and delivery of allogenic stem cells for cardiac tissue repair [12]. These titanium carbide‐based MXene quantum dots (< 10 nm) showed intrinsic immunomodulatory properties to reduce the activation of human CD4^+^IFN‐γ^+^ T‐lymphocytes while promoting the expansion of immunosuppressive CD4^+^CD25^+^FoxP3^+^ regulatory T‐cells in the stimulated lymphocyte populations. In 2021, as a follow‐up study by the same research group, the design and fabrication of another bioactive MXene composition, tantalum carbide quantum dots, using a hydrofluoric acid (HF)‐free protocol to suppress inflammation in vitro and prevent transplant organ rejection in vivo, and the treatment of cardiac allograft vasculopathy [15]. Through a detailed study, the authors proposed and tested the immunosuppressive functions of these new MXene‐based quantum dots and their underlying interactional mechanisms. It has been found that a one‐time treatment with these aqueous quantum‐dot dispersions could spontaneously regulate antigen‐presenting endothelial cells and alter their surface receptor expression to reduce the activation of allogeneic T‐lymphocytes and ameliorate the structural changes and early development of cardiac allograft vasculopathy. This study elucidated a high biocompatibility of the synthesized MXene quantum dots with immune cells. Furthermore, another research group has reported the first application of MXene nanosheets for antiviral and immunomodulation against SARS‐CoV‐2 [13]. These studies introduced the potential of rationally designed MXenes as future nano‐immunosuppressants for treating systemic/local inflammation and preventing organ rejection post‐transplantation, paving the way toward treating various inflammatory‐viral and autoimmune diseases [12, 13, 14, 15].

Moreover, in 2022, another follow‐up study reported the intrinsic in vivo immunosuppressive properties of titanium carbide MXene nanosheets [16]. The authors elucidated that these accordion‐like multilayer nanosheets could also effectively interact with human vein endothelial cells and downregulate the expression of alloantigen‐presentation genes, which contributes to reducing the excessive activation of allogeneic lymphocytes. Through transcriptomic analysis, they propose that treating with these nanosheets can selectively downregulate the corresponding genes in transplant‐induced T‐cell activation, cell‐mediated rejection, and subsequently cardiac allograft vasculopathy development. Their impact on reducing lymphocyte infiltration and preserving medial smooth muscle cell integrity within the transplanted aortic allografts has been shown. According to that data, a significant downregulation was also shown in several key antigen presentation mediators by HLA Class I, including interferon regulatory factor 1, transporter associated with antigen processing 1, and beta‐2 microglobulin. Additionally, a significant decrease was reported in the expression of cell adhesion molecules, including vascular endothelial (VE)‐cadherin and platelet endothelial cell adhesion molecule precursor 1, amongst the as‐treated endothelial cells with these MXene sheets, while the normal endothelial phenotype/function is maintained, including capacity for cytokine‐induced upregulation of leukocyte adhesion molecules upon early interaction with interferon‐γ [16].

The advances of this emerging field suggest the significant potential of immunosuppressive MXenes, opening up new avenues of study for the treatment of inflammatory and autoimmune diseases and specific viral infections. Building on these research findings, a recent study reported the application of MXene in preventing the rejection of transplanted cells after retinal degeneration in a mouse model [27]. This work presents the first in vivo proof‐of‐concept for MXene‐enhanced cell transplantation strategies, further confirming the previous findings on the impact of MXenes on in vitro stem cell delivery and in vivo prevention of transplant rejection. Using an innovative strategy, they have reported that ultrathin niobium carbide‐based MXene nanosheets can treat degenerative retinal disease in mice, confirmed by their impact on preventing progressive loss of retinal neurons. In particular, retinal progenitor cells were transplanted with and without applying in vivo MXene photothermal treatment and laser exposures in mice [27]. The goal was to address the currently existing challenges of low efficacy of transplanted retinal progenitor cells, which are largely limited by the imprecise neurogenic differentiation of these cells, and their underlying functions are affected by severe oxidative retinal lesions. These nanosheets have been shown to enhance the transplantation efficacy of these cells in tested models toward retinal regeneration. The MXene‐boosted photothermal treatment effectively promoted the retinal neuronal differentiation of the transplanted cells by activating intracellular signaling, along with spontaneous protection through scavenging of free radicals and generation of excessive reactive oxygen species. Their results suggested a dual function of these nanosheets, synergistically assisting the injected retinal progenitor cells at the host site, covering a promising paradigm in the rescue of retinal engineering for the future vision‐restoration field and nanomedicine therapies [27].

Taking these accounts into consideration, it can be concluded that specific compositions and forms of MXene possess intrinsic immunosuppressive properties, holding the therapeutic capacity to address the aforementioned inflammatory and pathogenic diseases. However, despite the progress obtained in this arena and the reported short‐term biocompatibility of MXenes at the tested dosages, critical concerns remain unclear due to the longer‐term safety and potential nanotoxicity or adverse metabolism effects and immunobiological pathways of intravenously injected novel materials into the bloodstream of research animals. Thus, this potential nanobiotechnology has remained dynamic with respect to further investigation on MXene synthesis processes, dose optimization, and enhancing their long‐term biocompatibility properties while retaining immunosuppressive functions.

In parallel with emerging universal investigations on extending the current knowledge of immunosuppressive profiles of MXenes and improving them to higher levels, designing highly rational and facile strategies that can be leveraged by existing literature is an additional asset. Given that this point is taken into consideration, pave the way toward how we can robustly rely on the use of current MXenes’ immunosuppression technology while significantly improving or reducing the associated biological risks. In this context, a key is developing route‐specific delivery methods for systemic immunosuppression, rather than direct injection of MXene dispersions into the bloodstream, which can deliver large‐surface‐to‐volume‐area particles into non‐specific and controlled systemic distribution. It is important to note that even though surface modification and dose optimization may, to some extent, reduce the potential adverse effects, their long‐term or undesirable sequential influences on immune cells and surrounding environments. Another crucial aspect is bioclearance with significant success rates, especially in the case of nondegradable MXenes (e.g., quantum dots) or those stronger‐bonded MXene nanosheets synthesized with lower surface defects and a tendency to decompose in aqueous/biological media. Nonetheless, hydrolysis gradually occurs at the MXenes’ structure with partial decomposition of their metal‐carbide bonds, releasing free transition‐metal atoms that are reactive to oxygen. This spontaneous reaction creates unstable phases of non‐bioactive metal‐oxide particles that, in addition to the existing trace of stable oxide compounds, may induce excessive production of reactive oxygen species (ROS), including hydrogen peroxide, ^•^OH radicals, and superoxide anions, contributing to cell death, apoptosis, and inflammation over time, hindering the immunosuppressive properties of MXenes. Thus, developing safer, route‐specific alternative administration strategies may enable more route‐specific delivery for sub‐site targeting of MXene to trigger indirect immunosuppression compared to direct interaction with blood compounds and circulatory accumulation in cells and organs, upon intravenous tail‐injections.

**FIGURE 1:**
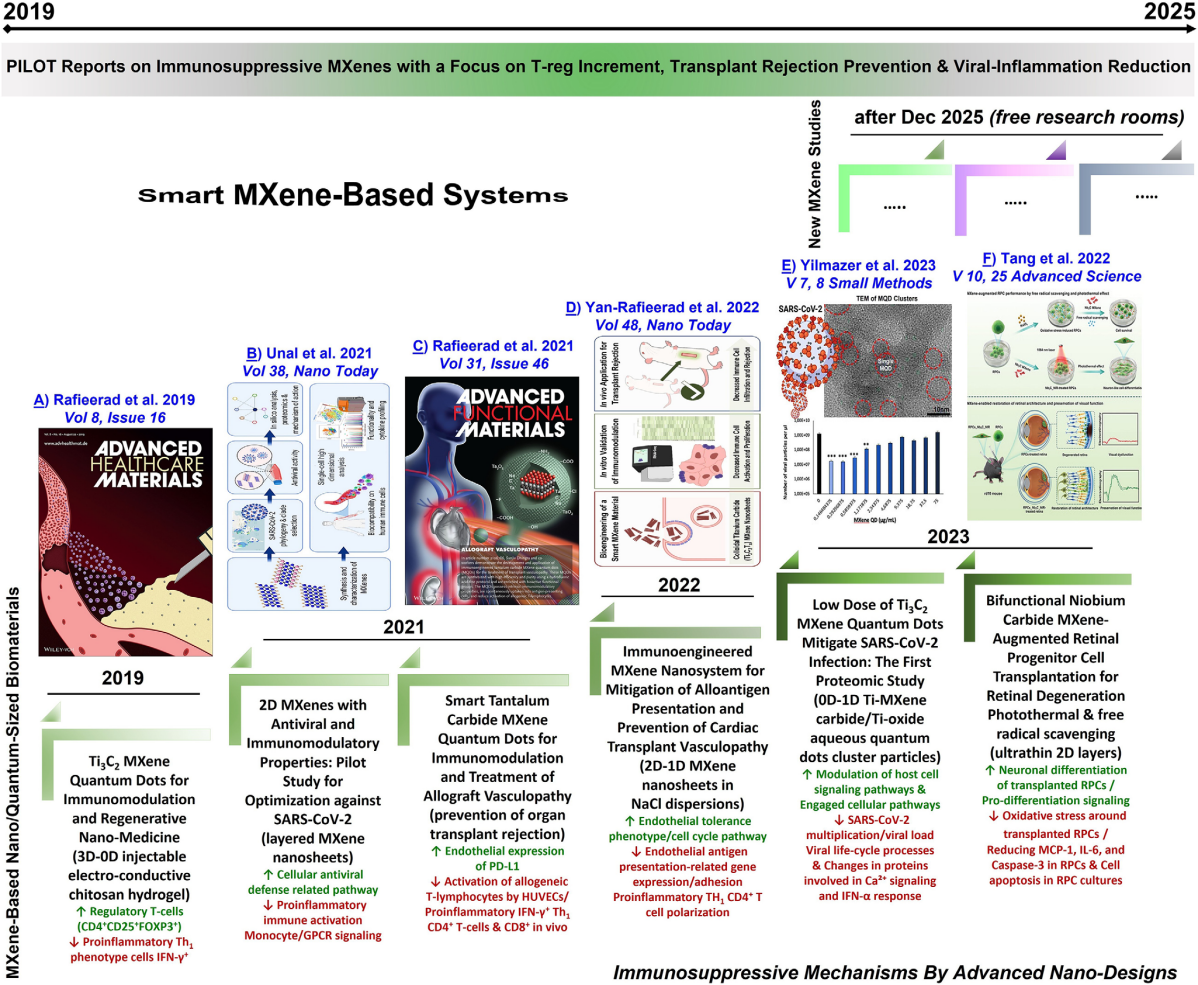
Representation of the reported pilot MXene‐based publications on immunosuppressive applications with a particular focus on increasing regulatory T‐cells, preventing donor transplant rejection, antiviral, and inflammation reduction. This roadmap (2019‐2025) on MXene‐enabled immunosuppression and their key mechanisms is schematically represented in a publication‐date dependent summary fashion, highlighting the potential of MXene biomaterials as next‐generation immunosuppressants. (A–F) Reproduced and merged with permission from refs [13, 15, 16, 20, 27, 28] respectively, Wiley/Elsevier, 2019, 2021, 2022, 2023, 2025, Open‐access copyright.

**FIGURE 2:**
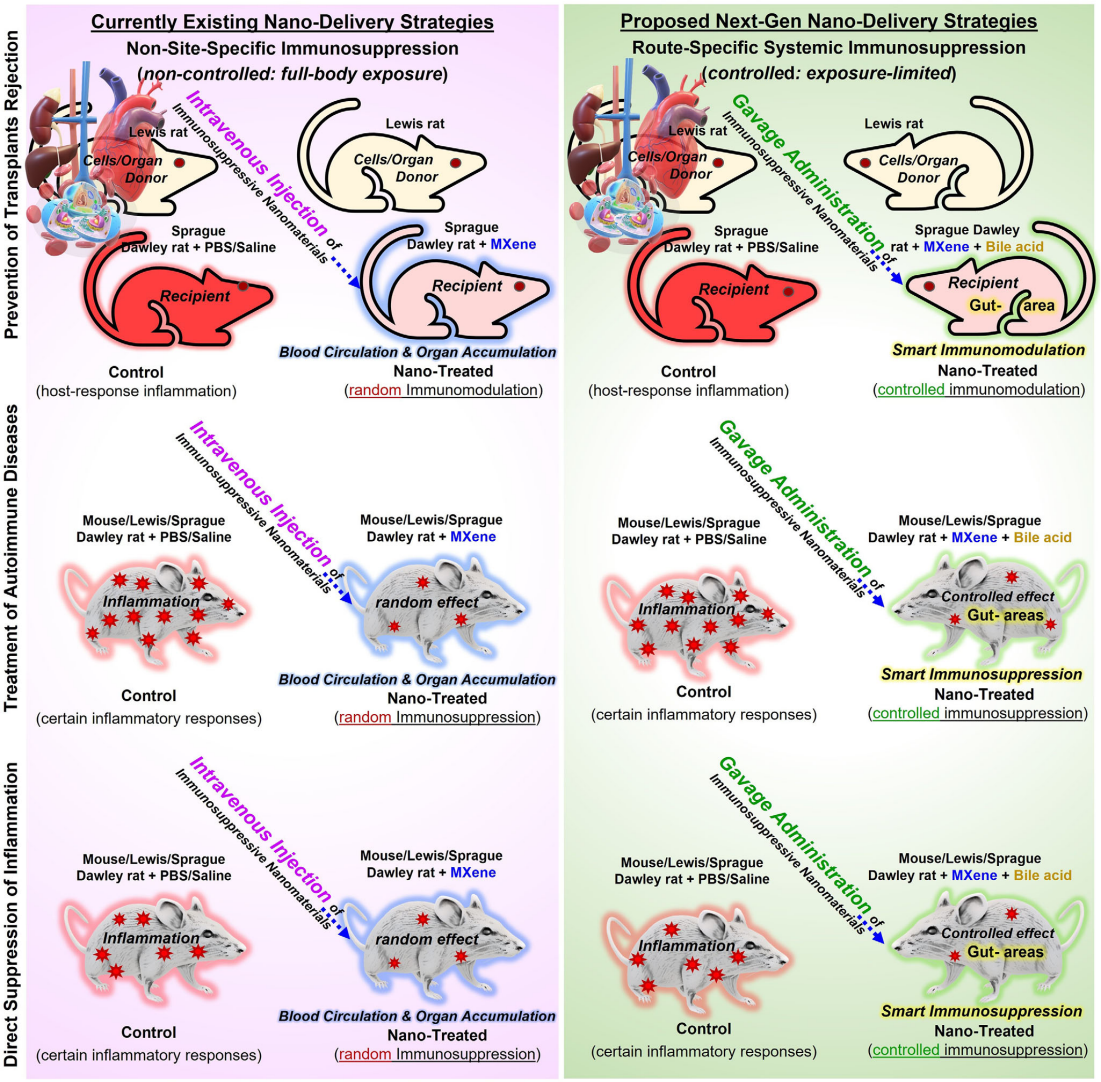
Illustration of schematic models of the currently existing common intravenous injection into the bloodstream of laboratory animals vs. the rationally proposed gavage administration strategies; expected safer immunosuppression in inflammation reduction and transplant rejection prevention, paving the way toward a route‐specific MXene immunosuppression strategy. The 2D/3D models and organs used in this figure are generated/customized from “Microsoft PowerPoint”.

In light of these considerations, we herein present an opinion‐based perspective on the rationale of the gavage‐administration method for delivering MXenes or other potential low‐dimensional immunosuppressive synthetic materials as an alternative to intravenous injection. We also proposed possible mechanisms of action of orally/gavage‐delivered nano‐dispersions to achieve intragastric delivery, gastrointestinal exposure, and subsequent interaction with immune cells and their underlying immunosuppressive signaling. Furthermore, novel bile acid surface‐modification strategies are presented to enhance the biocompatibility and nano bio‐interactions of gavaged particle dispersions with beneficial gut microbiomes and their subsequent clearance from the body. We also aimed to highlight recently uncovered immunosuppressive applications of MXenes by increasing Tregs and modulating the anti‐/pro‐inflammatory cytokines, serving as a roadmap for the design of further experiments based on pre‐established MXene compositions/forms. Current knowledge and roadmap journey of the most promising advances on immunosuppressive MXenes are shown in Figure 1. Additionally, an overview of the currently existing intravenous injection delivery strategies into laboratory animals vs. the proposed gavage administration methods and their anticipated lower risks for future treatment of inflammatory and autoimmune diseases, and prevention of donor transplant rejection, is schematically illustrated in Figure 2.

## 2 Significance, Potential, and Limitation of MXenes for Immunosuppression and Transplant Antirejection Applications

As referred earlier, recent studies reported the immune‐compatibility and activity of different MXenes at controlled doses and tested timepoints for inducing immunosuppressive mechanisms. These research advancements and other relevant works have significantly expanded the scope and boundaries of immunosuppressive MXenes in nanomedicine. It is therefore anticipated that widespread efforts are currently focused on further elucidating and optimizing the underlying immunomodulatory mechanisms, as well as improving the biocompatibility behaviors of MXenes.

**FIGURE 3:**
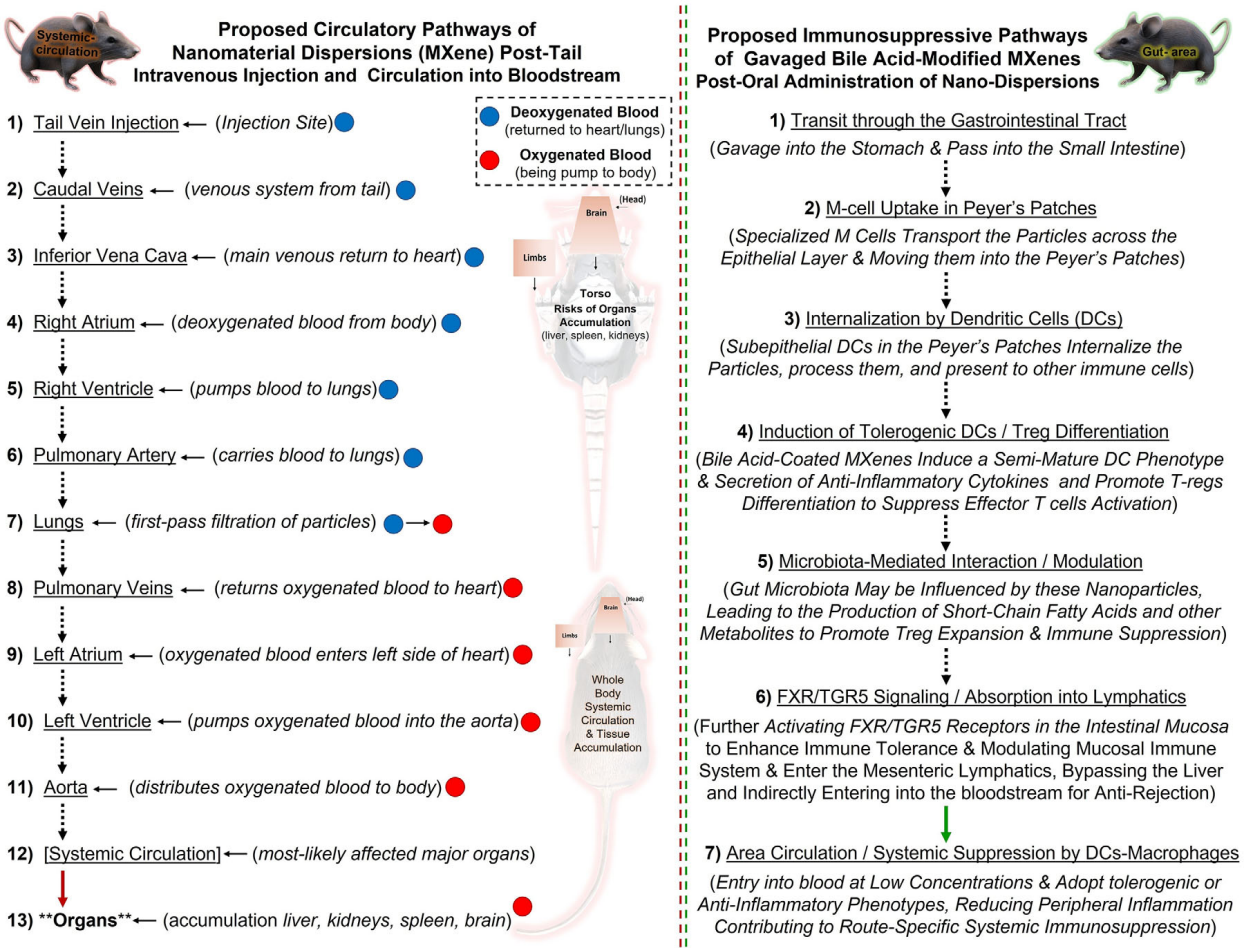
Illustration of the proposed distribution routes and in vivo interactions upon injection of engineered nanoparticles (e.g., MXenes) into the bloodstream of research animals and the gavage alternative immunosuppression pathway. The schematic model depicts the proposed general blood circulation map and potential nanotoxicity and adverse bio‐impacts on rats/mice upon intravenous injection, including systemic exposure, contact with blood immune cells, liver, spleen, kidney, and other organs vs. the proposed route‐specific gut‐area delivery. The 3D models in this figure are generated/customized from “Microsoft PowerPoint”.

Generally, when a synthetic nanomaterial, such as any chemical compositions of engineered flakes, sheets, particles, quantum dots, or derived heterostructures, is administered intravenously to the bloodstream of laboratory animals, blood circulation and direct delivery to circulatory or other organs, their surface interactions, particle distributions, or possible tissue accumulations are varied. This includes several factors related to the method/area of intravenous injection, delivered dosage, surface charge, and other physicochemical and biocompatibility properties of the injected nanomaterials. Figure 3 depicts an overview of the proposed in vivo distribution upon intravenous delivery and potential interactions of injected engineered nanoparticle dispersions (e.g., MXenes), as well as the gavage alternative immunosuppression pathway. Obviously, the biocompatibility of delivered particles at different cellular and organ levels is a key factor in determining their short‐term, mid‐, and long‐term safety or systemic in vivo nanotoxicity. Additionally, due to their direct circulation into the bloodstream and interaction with immune cells and other surrounding cellular complexes, including endothelial cells, fibroblasts, cardiomyocytes, osteoblasts, retinal cells, neurons, etc., potential risks also exist for metabolic perturbations or other associated adverse effects in the biological systems of intravenously treated animals.

To the best of this emerging field's knowledge, by injecting nano‐ or quantum‐scale surface‐bioactive particles into the small mammalian tail, such as rats or mice, immediate distribution into the bloodstream is certain. Spectacularly, the intravenous tail‐injected nanomaterials typically enter the interstitial spaces between the recipient animal's tissues and are eventually absorbed into their circulatory tissues/organs. This means that all the injected amounts of the given material enter the bloodstream and pass through the vessels and drain/engraft into organs, rather than being expelled into the gut or lymphatic system. Undesired thrombogenicity and particle accumulation in the serosa of the liver and spleen or double‐layer mesentery is another challenge of directly injecting particles into the peritoneal cavity. Thereby, their fate largely depends on several factors, including size, surface modifications, charge, and/or hydrophilicity. Typically, small particles (≤100 nm) are more likely to be distributed to various major organs, such as the heart, kidneys, spleen, lungs, and even sometimes the brain, if they cross the blood‐brain barrier. In the case of larger particles (≥100 nm to 5–10 µm), they may be trapped in the spleen and liver due to the reticuloendothelial system, acting as a spontaneous biological filter. Depending on their sizes, the distributed synthetic particles can be partially and potentially eliminated from the body through different bio‐clearance routes. One of the most likely scenarios is through extraction via the kidneys. In particular, smaller‐sized particles that are not absorbed by the interacting tissues might get filtered in the kidneys and excreted from the body through urine. The ultrasmall particles (≤10 or 20 nm) may also be cleared from the body through renal filtration.

Another likely body mechanism for the extraction of foreign engineered nanoparticles would be through the liver. Larger particles (and sometimes also those that are phagocytosed by immune cell macrophages) can be further processed in the liver, subsequently followed by being excreted in bile and/or undergoing catabolic activities. Fecal excretion is another possible mechanism for clearing the injected nanomaterials after ingestion or entry into the lymphatic system via the gut. Thereby, the particles could eventually reach the gastrointestinal tract and be excreted from the animal's body through feces. However, the actual feasibility of this method is largely limited to injected nanoparticles, as vein administration routes are less common than can end up in the digestive system through indirect pathways. In fact, gut involvement is not a primary exposure route for intravenously or subcutaneously injected particles unless they enter the lymphatic system or are ingested. Even if this scenario works with low efficiency, it can lead to excessive collection of fat‐soluble substances from the intestine, which may not be ideal under normal conditions. Intravenously‐injected materials do not typically and directly enter the gut sites, unless the given particles undergo rare possible endocytosis processes for intravenous treatments, such as by gut‐associated immune cells, or if there are leakages within the vascular barrier, which is uncommon.

Less likely, more elongated bioaccumulation durations of the injected nanomaterials in the liver, spleen, or lungs may also be affected, depending on their size, form, surface topography, pH, and charge properties. This distribution tendency of ultrasmall bioactive materials into specific targeting cells or organs, such as tumor sites in cancer therapies, may be beneficial for their delivery/tracking in particular tissue types or immune‐related organs in immunomodulatory applications. However, in a broader picture of biological perspectives, long‐term cellular localization or persistent uptake and tissue accumulation, in which injected particles are largely retained within cells/tissues for periods longer than required for their intended function, are generally not considered desirable for immunosuppression in practical therapeutic strategies. Besides, if suppressing the immune cells is the ultimate target of the injection of engineered nanomaterials, a clear understanding of their interactions with macrophages, dendritic cells, and/or T‐cells, at different timepoints is certain, as these cells reside in major organs: in the liver, predominantly throughout Kupffer cells, and in the spleen, where they often involve in immune responses) under inflammatory and non‐inflammatory conditions. This may justify the popularity and efficiency of vein delivery methods for injecting immunosuppressive nanomaterials, including MXenes, in related publications. Nonetheless, the concerns regarding the long‐term in vivo toxicity and adverse metabolic or immunological effects of novel nanomaterials remained unsolved. These challenges further underscore the importance of assessing the feasibility, safety, and efficacy of next‐generation delivery methods for targeted application with a lower risk and more efficient bioclearance from the body after their enrollment as immunosuppressive agents. Therefore, applying safer and more feasible alternatives is at the forefront of the field to reduce the risk of direct blood circulation rather than being flushed out through the gut, while still benefiting from their intrinsic immunosuppressive properties.

In accordance with the proposed route‐specific gavage immunosuppression pathways in this perspective, it is also anticipated that oral administration of engineered nanomaterials, including bile acid‐modified MXenes, offers advantages over direct intravenous injection into the bloodstream. This encompasses indirect circulation through lymph, and macrophage modulation, local induction for Treg expansion, positive interaction with gut microbiota, and lower/modulated systemic exposure of particles, reducing the risks of high peak concentrations, toxicity, and tissue accumulation. Moreover, from pharmacokinetic and biodistribution perspectives, gavage delivery may enable sustained local and semi‐systemic mucosal immune programming compared to prompt systemic exposure. Further, lower risks of off‐target tissue accumulation (liver, spleen, kidney) or metabolic shock are expected as particles predominantly interact with the gut/lymph at early stages.

### 2.1 Alternative Gavage Strategies for Immunosuppressive Nanomaterials Delivery

From a medical perspective, oral administration of immunosuppressive or anti‐inflammatory drugs is a common approach for recipients of donor cells/organ transplants during the early stages or long‐term prescriptions for patients with autoimmune or chronic inflammatory diseases. Indeed, administering medications directly into the stomach or via route‐specific gavage administration into the gut, and subsequently passing through intestinal membranes and the lymphatic circulation, may be a beneficial method for multiple or long‐term treatments due to its ease of administration, fewer side effect risks, and slower immunosuppressive mechanisms of action. Additionally, the nano‐dispersions’ pass‐through digestion process may benefit from sustained release and route‐specific interactions with immune cells and underlying signaling pathways to modulate systemic immunosuppression and antirejection effects on donor transplants. Besides, due to limited direct contact with the bloodstream and subsequent tissue accumulation, reduced physical damage attributable to exposure to gavage‐administered particles with increased bioclearance is proposed. This rationale supports testing the efficacy of gavaging of immunosuppressive nanomaterials.

Obviously, several accounts need to be taken into consideration to validate and optimize this strategy. First, immunosuppressive nanoparticles must be surface‐modified to enhance their overall biocompatibility properties. The second challenge is studying the detailed interaction of gavaged nanoparticles with beneficial gut bacteria. Lastly, the treatment and interaction must undergo under the actual gut's physiological and biochemical conditions to evaluate the efficacy of the proposed method under in vivo digestion mechanisms, in situ degradation, and oxidation of surface‐modified nano‐dispersions, along with thoroughly studying their immunosuppressive roles and bioclearance. Indeed, none of these essential requirements is currently explored, especially for MXenes. In addition, bile acid modification, which may not only improve their immunosuppressive properties due to their immunomodulatory nature, but also can enhance their biocompatibility with gut's beneficial microbiomes to be somehow identified these bile‐acid‐coated particles as “self” and ease their transportation to intestinal membranes for effective interaction with distinct immune cells and specialized epithelial cells (M cells) to transport antigens from the lumens to dendritic cells and lymphocytes, and subsequently enrolling them into the digestion process for significant flush out from the body. In this perspective, we offer for the first time a facile and multifunctional bile acid surface modification strategy to enhance the biocompatibility and potentially immunosuppressive properties of MXenes. If this new concept can be experimentally evidenced, it would be a field's breakthrough, providing a next‐level delivery route of immunosuppressive agents with lower nanotoxicity risks, paving the way for future practical optimizations and pre‐clinical research.

## 3 Further Discussions on the Rational Behind the Proposed Hypothesis and Its Feasibility

In accordance with the knowledge gaps and problem statements explained in the previous section, the ultimate goal proposed in this perspective is to rationally surface modified MXenes, and potentially other related immunosuppressive nanoparticles, contributing to facilitating their bioclearance pathways through more route‐specific deliveries. In particular, the hypothesis turns around the idea that rather than the particles being mainly filtered/accumulated by the kidneys and liver, they can be directed to the gut for immune regulation and subsequently flushed out from the body. Since the entire digestion process often takes 24–72 h (considered a relatively quick process), two or multiple gavage treatments may be required for desirable immunosuppression and antirejection responses. While multistage treatment can be challenging, it might be worth reducing the risks of blood contamination, systemic toxicity, and non‐specific tissue accumulation. The key aspect of this scenario is how to modify immunosuppressive MXenes to efficiently enhance their interaction with the gut and lymphatic systems to direct them into the gastrointestinal tract, which is likely to be excreted through urine and feces.

For the success of the proposed scenario, several approaches need to be thoroughly explored through detailed mechanistic studies. Even though modifying the nanoparticles’ design or delivery method can facilitate their transport to the gastrointestinal tract via an indirect gut route, the direct pass route through the intestinal epithelium and into the gut‐associated lymphoid tissue remains considerable. While the fundamental physicochemical properties‐dependent behavior of MXenes under gastrointestinal conditions and actual gut environments are being investigated with limited frequency, there is concurrent research into exploiting their tunable biodegradation based on the targeted applications in oral drug delivery or the treatment of inflammatory bowel disease therapies. Rational surface modification of MXenes and incorporating them into biodegradable composites are at the forefront of research in this field. The zeta phonetical data suggested a negative charge of MXenes (e.g., tantalum carbide) at different pH levels ranging from 2 to 11, suggesting their relative stability and surface characteristics of MXene functional groups under distinct acidic and mildly basic conditions [15, 29]. Based on the literature, the effect of MXenes’ physics and chemistry under gastrointestinal and non‐simulated gut conditions (pH ∼1.5‐3.5, ∼5‐5.5 in the large intestine, and up to 6–6.5 in the duodenum small intestine), is limited in assessing their stability or short‐term toxicity release [30, 31, 32, 33]. These studies reported that MXenes, due to their tunable physicochemical properties, exhibit variable stability behavior in the gastrointestinal tract, ranging from degradation in highly acidic environments to improved stability in drug delivery applications. It is likely that with the involvement of the liver, MXenes be degraded within the gastrointestinal tract for specific applications or programmed for gastric degradation resistance through integration into clinically relevant biodegradable and water‐swollen hydrogels, such as collagen, gelatin, and hyaluronic acid, for controlled release and sustained immunosuppression.

In this context, functionalization of engineered particles with lipophilic coatings, such as fatty acids, phospholipids, or other relevant biodegradable compounds, may also support their passing through intestinal membranes more efficiently and entering the lymphatic circulation. The oral delivery of water/saline‐dispersible nanomaterials rather than intravenous injection can not only direct high surface‐area bioactive particles to the gut areas, where they can be digested, absorbed, or excreted, but also reduce random distribution in the body. Enteric protective biocompatible and active coatings may potentially prevent their prompt degradation in the acidic gastric environment. This enables the gavaged nanoparticles to release their bioactive contents and engage in surface interactions for immunosuppressive signaling upon reaching the alkaline environment of the small intestine. For these particles to enter the intestine, if sufficiently small, they may be partially absorbed/transferred into the bloodstream, which can promote their immunosuppressive functions. In the case of larger particles and those small‐sized traces of particles that are not being absorbed, excretion via the feces is the most likely sequence. This further highlights the gavage application to evade massive blood circulation involvement.

It is anticipated that MXene particles with a size of less than 1 µm are more likely to interact with the gut mucosal surfaces and/or be retained in the gastrointestinal tract. Larger traces may remain in the gut and eventually be excreted. Surface functionalization with biopolymers, such as chitosan, polyethylene glycol, and dextran, can, to some extent, modify MXene dispersions with a better stability in the gastrointestinal tract and interaction with specific target receptors on the gut's epithelial cells. In addition, gavage delivery of immunosuppressive nanoparticles may benefit from the lack of direct interaction with the mouth (tongue and gums) compared to the route to the lymphatic system during oral mucosal delivery. It may also offer a lower risk of particle entry into the systemic circulation via lymphatic drainage as compared to the buccal mucosa or sublingual tissues. The approach relies on the desired bioactivity of MXene dispersions to interact with diverse cells in the gut for effective immunosuppression and bioclearance. Moreover, oral administration of immunosuppressive and anti‐inflammatory drugs can be combined with gavage application of MXenes to enhance the efficacy of hybrid methods at early stages of transplantations and reduce the side effects of long‐term therapies. The effectiveness depends on various factors, including physicochemical behavior of bile acid‐coated MXenes under gastrointestinal conditions at different of gavege delivery stages. Another effective key parameter could be pH‐dependent biodegradation, in situ oxidation, enzymatic exposure, particle aggregation, and changes in their surface functional groups in gastric/intestinal environments within the duration of treatment as described accordingly.

The first mechanism of action is centered on gut‐associated lymphoid tissues, as the gut is home to a large part of the immune system, including these tissues, containing Peyer's patches, M cells, and other lymphoid structures. When orally given nanoparticles are ingested, they can subsequently interact more safely with the immune cells in the gut, induce immune tolerance signaling pathways, or directly suppress inflammation and prevent transplant rejections. This rational speculation further highlights the gavage administration and gut in situ target of bioactive MXenes for immunomodulation. Moreover, certain nanoparticles, such as micelles or liposomes, can also be functionalized with MXenes to induce immunosuppressive responses. Specific antigens can also be loaded to boost the induced tolerance and more effectively suppress immune activation, particularly for controlling autoimmune diseases and preventing donor cell/organ rejection. This strategy may potentially reduce the needed doses of drugs or the required number of immunosuppressive gavage treatments. Optimal formulations of modified MXenes or synergistic MXene‐drug hybrid methods, including oral immunosuppressant drugs used for donor cells/organ transplants, inflammation, and autoimmune diseases, may enable more efficient release, leading to prolonged inflammation suppression, especially in transplantation cases, where extended periods of immune modulation are required.

Notably, the gavage or oral administration of engineered immunosuppressive nanoparticles may encounter limitations. The most challenging aspect might refer to absorption and distribution, highlighting the necessity of determining the optimal size and dose ranges of MXenes. The desired particles are neither excessively small nor too large to be efficiently absorbed through the intestinal epithelium and subsequently transported into systemic circulation, and/or be excreted in feces. The key is manipulating the time required for the gavaged particles to play their suppressive roles, while they do not fully absorb into the bloodstream to be easily flushed out from the gut and the body. It is also notable that the subcutaneous, intravenous, or intramuscular injection can be considered in scenarios where rapid systemic distribution of immunosuppressive agents is critical. For instance, if organ rejection is imminent or acute immunosuppression is observed after transplantation, direct injection can be provided for faster effects and a more controlled and route‐specific administration. This theory is not contradictory with the rationale behind the proposed gavage administration of engineered particles, as in the case of transplanting smaller organs like the kidney, and upon injecting allogenic stem cells and certain autoimmune disease conditions with less T‐cell activation, where the medical urgency is not on the priority list, and gavage can still be potentially beneficial. Besides, the dosage and frequency of the treatment are not expected to differ significantly between intravenous and gavage administration, if MXene biomaterials are optimally fabricated and surface modified. Thus, DNA damage, chromosomal alterations, and genotoxicity can be reduced.

### 3.1 Bile Acids Coating: Key Benefits and Potential Bioactivity Enhancements

A rational, facile, and biocompatible surface‐modification strategy to enhance the feasibility of gavage administration of hydrophilic nanomaterials (e.g., MXene) or hydrophobic immunosuppressive particles is an asset. Indeed, coating of dispersions with bile acid or its derivatives can positively impact the gavage treatment outcomes. The proposed oral administration must not only effectively induce immunosuppressive mechanisms, but also be compatible with digestive enzymes and the gut‐beneficial probiotics. Indeed, bile acids are naturally occurring amphipathic molecules that contain both lipophilic and hydrophilic regions produced by the liver to help with the digestion and absorption of fats. Potential advantages of bile acid coating in the context of gut‐targeted delivery encompass high biocompatibility with the gut environment, mimicking natural interaction between bile salts and gut microbiota and intestinal epithelial cells to enhance absorption and synergistically support sustained immunosuppressive responses.

Furthermore, bile acids possess the intrinsic ability to modulate the gut microbiomes. Indeed, they are essential for the growth of certain probiotics in the gut and also pivotal in their maintenance and biological reproduction processes. In particular, most lactic acid bacteria (e.g., *Lactobacillus* or *Bifidobacterium*) tolerate certain amounts of bile acids, while others respond differently and may be inhibited by high concentrations of bile. The idea of surface coating immunosuppressive MXenes with proper bile acid types/doses contributes a dual role in promoting the growth of gut's beneficial microbes and protecting them from the acidic conditions in the stomach, bile salts in the intestines, and preventing rapid digestion. This strategy may also support their safe transport to the specific regions in the gut, such as the colon or ileum, for enhanced bioefficacy and bioclearance.

Moreover, bile acids have a significant biological role in emulsifying fats and increasing the solubility of hydrophobic compounds/substances. As‐synthesized MXene nanosheets are mostly hydrophilic and not inherently lipophilic (oil‐loving); however, their water dispersibility needs to be improved. Coating MXenes with an optimal dosage/type of bile acids under specific conditions may enhance their dispersion properties in water, allowing for a higher absorption across the intestinal epithelial barrier. Surface modification with bile acids may also potentially protect MXene particles from prompt degradation by intestinal enzymes in the stomach's environment, acting like a biocompatible lipid coating. This approach may also influence synergistic effects on gut microbiota toward immune modulation, metabolism, and inflammation suppression, which can enhance the immunosuppressive roles of MXene after transplantation. Bile acids interact with signaling molecules through their related receptors, including Takeda G protein‐coupled receptor 5 (TGR5) and Farnesoid X receptor (FXR). Together, bile acid‐coated MXenes have the capacity to modulate immune responses by regulating intestinal inflammation and immune tolerance for the treatment of inflammatory/autoimmune diseases and preventing the rejection of early‐stage organ transplants. Possible challenges and risk factor considerations must also be studied. This includes possible toxicity at high doses, over‐interaction or uncontrolled impact on gut microbiota (growth, proliferation, survival), inefficient coating outputs, and unencapsulated release in the small intestine or colon. Since bile acids are naturally occurring amphipathic molecules produced by the liver, it is speculated that optimal bile coating of MXene would be advantageous with negligible adverse effects, which must be examined and validated robustly by experiments. This note highlights the superior potential of bile acids over lipid‐based coatings for improving the gavage‐administration theory of MXenes for immunosuppression. Accordingly, we discuss the most certain bile acid and facile attachment or surface binding to both types of hydrophilic and hydrophobic nanomaterials. This enhances the feasibility of the gavage administration method for next‐gen MXene‐enabled immunosuppression.

### 3.2 Significance of Coating MXenes with Cholic Acid, Deoxycholic Acid, or Taurocholic Acid and Proposed Interaction with Immune Cells and Gut Microbiota for Route‐Specific Immune Modulation

Surface coating of engineered nanoparticles with bile acids can fundamentally enhance their biocompatibility, intestinal residence and uptake, microbiota‐mediated transformation processes, and, under certain condition also contribute to modulate the host‐microbe signaling, like nuclear farnesoid X receptor (FXR) and the membrane Takeda G‐protein receptor 5 (TGR5), reducing nuclear factor kappa B (NF‐κB) signaling and nonspecific immune activation. Given that, the key point is which bile acids can most effectively coat MXenes in a way that not only enhances their immunosuppressive and dispersibility properties, but also facilitates gavage treatment and interactions with gut microbiota. It is known that specific bile acids, including cholic acid and chenodeoxycholic acid (a primary type synthesized in the liver and secreted into the intestine, where it interacts with the gut beneficial microorganisms/receptors), possess immunosuppressive and anti‐inflammatory activities to inhibit lymphocyte over proliferation and immune activation. These compounds are highly potent and can also be used as an alternative to lipidic coating on the surface of gavaged‐MXene or other immunosuppressive particles, acting as a protective biolayer against prompt degradation, potential nano‐toxicity, and promoting bio‐excretion. Cholic acid‐based components may also contribute to improving gut digestion and support beneficial bacteria in the gut, considering their high biocompatibility, gut‐friendly nature, and beneficial impacts on digestion/clearance of synthetic nanoparticles from the body. This significance is more highlighted when multiple gavage administrations (or even in conventional intravenous injection into the bloodstream) are required at early stages after organ transplantation or in severe autoimmune and inflammatory disease treatments. In this regard, several commercially available compounds could be purchased for coating of nanoparticles intended for future gavage administration or oral delivery.

**FIGURE 4:**
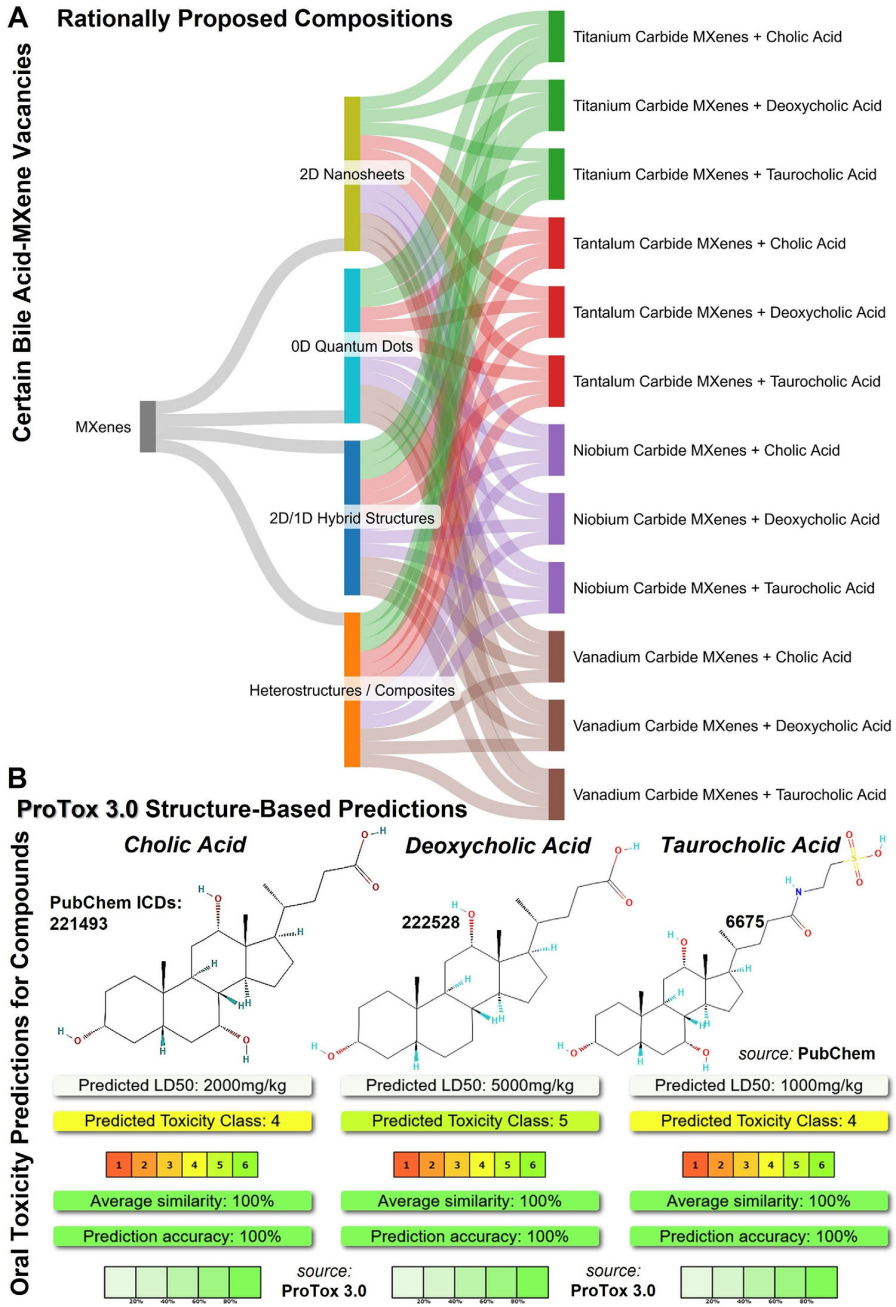
(A), The tree probability diagram of MXenes of different compositions/dimensions/forms, as well as potential bile acid coating for gavage‐delivery immunosuppression applications. This figure presents the workflow of the rationally proposed future experiments by giving equal probability to each MXene/bile acid for fair presentation (a total of 52 flows with frequency of parent to new nodes, summation of all components’ probability in cluster ≈1.0). It was made at “SankeyMATIC” Open Diagram web tool by Steve Bogart (https://sankeymatic.com/). (B), the 2D chemical structures of distinct bile acids (PubChem https://pubchem.ncbi.nlm.nih.gov/), including cholic acid C_24_H_40_O_5_, MW: 408.6 g/mol, CID:221493, deoxycholic acid C_24_H_40_O_4_, MW: 392.6 g/mol, CID:222528, and taurocholic acid C_26_H_45_NO_7_S, MW:515.7 g/mol, ID: 6675. It also depicts the “ProTox‐3.0” oral toxicity prediction (LD50 volumes & class ranges) of the input compounds, suggesting a high level of their overall compatibility with biosystems at the recommended doses. The screenshots of the toxicity chart (quick result) of these bile acids compared to the average of their class with reference to a paper by Priyanka Banerjee et al. , ProTox 3.0: a webserver for the prediction of toxicity of chemicals, *Nucleic Acids Research*, V 52, I W1, 5 J 2024, P W513‐W520 (https://tox.charite.de/).

Additionally, the deoxycholic acid or taurocholic acid are other potential bile acids that can be considered, depending on the targeted immunosuppression, working sites, or the importance of gut microbiome modulation. Their key structural difference is that cholic acid is a primary bile acid used in lipid emulsions, whereas deoxycholic acid is a secondary bile acid that can also affect the gut microbiota and is often considered in liposomal formulations for targeted delivery and controlled release into biological environments. It is anticipated that coating immunosuppressive nanomaterials with bile acids may differentially modulate immune responses when administered orally. In particular, due to diverse chemical compositions/forms of MXenes, the superiority of each bile acid‐coated MXene design must be carefully tested under the same experimental conditions. Theoretically, it can be hypothesized that modifying particles with cholic acid may more effectively enable microbiota‐compatible anti‐inflammatory effects, while chenodeoxycholic acid coating may better induce FXR‐mediated immunosuppression. In the case of deoxycholic and taurocholic acids, the most likely scenario is to elicit dose‐sensitive‐TGR5‐dependent immune regulation and support microbiota‐responsive and barrier‐protective interactions, respectively. Table 1 proposed a comparison of the immunosuppressive properties and key beneficial gut interactions of surface coating with distinct bile acids. Further, Figure 4 presents the probability of different MXene‐bile acid designs and the biocompatibility rate/dose prediction of bile acids.

**TABLE 1:** The proposed comparison of the immunosuppressive properties and key beneficial gut interactions of nano‐surface coating of MXenes with potential bile acids.

Specifications	Cholic acid	Chenodeoxycholic acid	Deoxycholic acid	Taurocholic acid
Class of bile acid	Primary class	Primary class	Secondary class	Conjugated class
Key immunosuppressive effect (gut area)	Mild to moderate	Moderate to high	High (dose‐dependently)	Mild
Main immunomodulatory pathway	Barrier protection, mild NF‐κB inhibition	Strong FXR‐mediated anti‐inflammatory signaling	TGR5‐driven cyclic AMP signaling	Indirect (through microbial deconjugation)
Impact on pro‐inflammatory cytokines	–↓ TNF‐α & IL‐6	–↓ TNF‐α, IL‐1β, & IL‐6	–↓ TNF‐α & IL‐12	Indirect reduction
Compatibility with beneficial microbiota	High	Moderate	Low to moderate	High
Gut barrier support	High	High	Moderate	High to very high
Microbiota‐responsive behavior	Moderate	Moderate to high	Low	High (dependent to bile salt hydrolase)
Suitability for chronic inflammation	High	High	Moderate (limited)	High to very high
Overall gut safety rate	High	Moderate to high	Moderate	High to very high
Microbiota disruption	Low	Moderate (dose‐dependent)	High	High to very high

Moreover, biocompatible polysaccharides and natural polymers, such as chitosan or alginate, may be suitable agents for MXenes, providing a protective layer against excessive oxidation and prompt degradation for longer biocompatibility and efficient immune suppression and bioclearance. They may also play a positive synergetic effect on interacting with the gut microbiome. Specific polyphenols and natural products can also be considered for MXene coating and modulatory roles in regulating gut microbiota due to their inherent antioxidant properties that can benefit the gut lining. Tannic acid is an example. The hydrophobic tail of bile acids and other related compounds can readily interact with the surface of active nanoparticles, while their hydrophilic parts (hydroxyl groups and steroidal backbone) can promote the stable dispersibility of immunosuppressive nanomaterials in aqueous/biological/physiological conditions. Surface of synthetic nanomaterials, depending on their hydrophilicity or hydrophobicity, can also undergo surface/hydrogen bindings to conjugate bile acids or biopolymeric compounds with MXenes. Physiological levels and bile acid dosages need to be prescribed by animal surgery experts or medical/pharmacological scientists.

### 3.3 Density Functional Theory (DFT) Calculations on Proposed Chemical Interactions of Bile Acid with MXenes for Surface Modification

**FIGURE 5:**
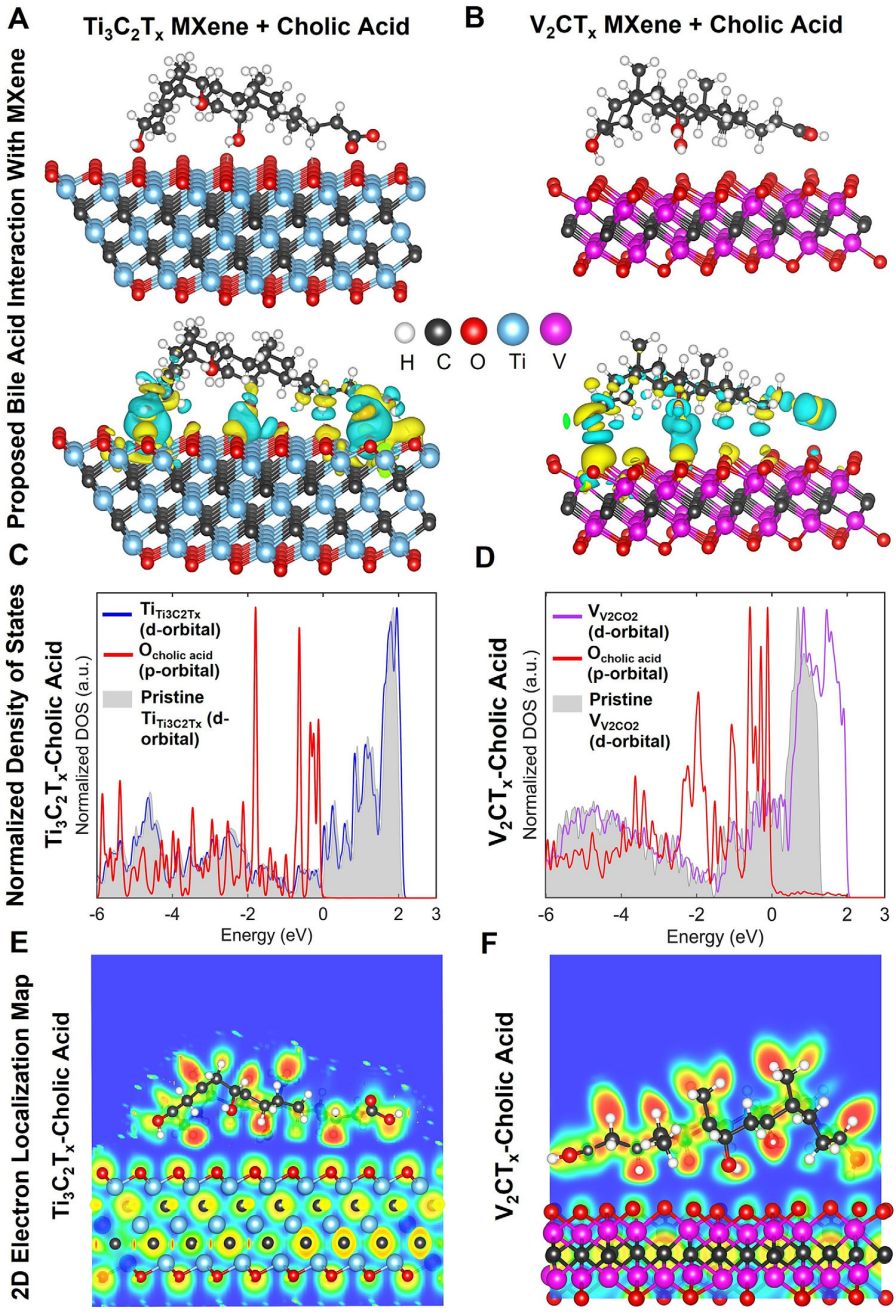
(A,B), Optimized adsorption configurations of cholic acid on Ti_3_C_2_T_x_ and V_2_CO_2_ MXene surfaces. The top panels show the relaxed atomic structures of cholic acid adsorbed on the surface of these MXenes. It depicts the corresponding charge density difference isosurfaces, where yellow and cyan regions denote electron accumulation and depletion, respectively. The normalized partial density of states of the corresponding systems, with shaded regions representing pristine MXene states and colored lines indicating contributions from transition‐metal “d” orbitals and cholic acid oxygen “p” orbitals, is shown in panels (C–F). Bottom panels show 2D electron localization function maps of the respective systems.

To obtain the molecular‐level understanding of the proposed interaction between MXene and representative bile acid (i.e., cholic acid) and their chemical stability at simulated body temperature (∼37°), we performed DFT calculations with the Vienna Ab initio Simulation Package (VASP). As shown in Figure 5, one cholic acid molecule was separately placed on the surfaces of two representative immunosuppressive compositions of MXene (Ti_3_C_2_T_x_ and V_2_CO_2_, including surface oxides). First, the simulated hybrid systems are optimized by geometry relaxation until the forces on all unconstrained atoms are below the chosen convergence threshold (10^−5^). Accordingly, the optimized systems are used to analyze charge density difference, density of states, and electron localization functions to understand the nature of the interfacial bonding between cholic acid and MXenes. For all these calculations, the projector augmented wave method and the Perdew‐Burke Ernzerhof exchange‐correlation functional are utilized. The long‐range dispersion interactions are calculated by the DFT‐D3 method. In‐plane, periodic Ti_3_C_2_T_x_ models and V_2_CO_2_ are built with periodic lengths in the in‐plane direction and a vacuum layer of at least 15 Å long along the normal plane. Cholic acid molecules are deposited on the surface of MXenes, and geometry optimizations are performed to relax all the atoms in the molecules and hold the MXenes slab in place until the forces have dropped to less than 0.02 eVA^−1^. The cutoff energy of a plane wave is set to 520 eV, and the Monkhorst–Pack k‐point mesh is scalable to 2D systems. The electronic structures of these systems are analyzed on the optimized structures, including the projected density of states, charge density difference, and electron localization function. Moreover, the interfacial charge redistribution is visualized by obtaining charge density difference maps that were calculated by the difference between the charge density of the isolated MXene and cholic acid, and the system.

Figure 5A,B shows chargedensitydifference (CDD) isosurfaces of cholic acid molecule on Ti_3_C_2_T_x_ and V_2_CO_2_, respectively. The electron accumulation (yellow) and depletion (cyan) at both modeled CA‐MXene interfaces are relatively localized. On Ti_3_C_2_T_x_, the charge difference is localized near the −O/−OH terminals on the surface and hydroxyl and carboxyl groups of cholic acid. A similar trend has been reported for the rearrangement of charges witnessed during the reaction of SO_2_ with cobalt‐terminated CoP surfaces, where electrons are transferred to the adsorbate on the surface Co atoms, leaving behind accumulation lobes between the oxygen atoms and the metal [34]. The CDD characteristics of the other MXene revealed a similar localized nature around the cholic acid oxygen atoms, and the uppermost V−O layer, indicating that the interaction between them is not strong chemisorption but may occur through hydrogen bonding and electrostatic polarization. It is known that this electronic signature is typical of non‐covalent adsorption in which the interaction is due to hydrogen bonding and electrostatic polarization, as opposed to orbital hybridization or charge transfer bond formation.35 In addition, patterns of similar charge‐density have been observed in the case of physisorbed organic molecules on metal oxides as well as 2D materials, where interfacial stabilization is predominantly through hydrogen bonding, and interactions between dipole and surface interactions [35, 36]. The similar trend in the distribution of charge on Ti_3_C_2_T_x_ and V_2_CO_2_ suggests that the surface termination chemistry, and not the specific transition metal, is the most relevant in the process of catalyzing bile acid adsorption, which is a possible interaction. Panels C and D in this figure exhibit the normalized partial density of states for cholic acid adsorbed on these MXenes. The shaded region corresponds to the pristine MXenes density of states without the cholic acid, while the colored lines represent “d” orbitals of titanium and vanadium atoms in their chemical structures, and the “p” orbital of the oxygen atoms in cholic acid. In both models, the transition metal ‘d’ states are dominant in the electronic structure in the energy range of about –6 to –1 eV, which is also typical of metallic MXenes [37]. On the other hand, the oxygen ‘p’ states of cholic acid occur mainly in the deeper region of the valence, featuring an insignificant part in the Fermi level. Notably, there are no new electronic states formed at the Fermi level or close to it, or a large‐scale overlap between the metal “d” states and cholic acid ‘p’ states near the Fermi energy. The lack of these hybridized features implies that there is no orbital mixing and covalent bond formation between the bile acid molecule and these MXenes’ surface in the adsorption process [38]. It is, therefore, anticipated that cholic acid forms noncovalent complexes with Ti_3_C_2_T_x_ and V_2_CO_2_ surfaces. This behavior is similar to the non‐covalent decoration of Ti_3_C_2_T_x_ channels with ethanol, in which a stabilization of hydrogen‐bonded solvent molecules within the nanoconfined interlayer space, without any significant disruption of the band structure of MXenes, has been previously reported [39].

Lastly, Figure 5E,F presents the 2D electron localization function (ELF) maps of cholic acid adsorbed on these MXenes. High ELF values (red regions) represent strongly localized electrons, such as covalent bonds and lone pairs, intermediate (yellow‐green) and low (blue) values represent more delocalized electron density. In both of these modeled systems, high ELF values are restricted to the internal C–C, C–H, and C–O bonds of the cholic acid molecule and M–C and M–O bonds of the MXenes’ frameworks. At cholic acid‐MXene interfaces, the ELF is consistently in the green‐blue range without continuous red bridges between cholic acid oxygen atoms and titanium or vanadium atoms on the surface. These ELF patterns are typical of weakly covalent or non‐bonding interactions and in sharp contrast to the high degree of covalent bonding that can be seen for arylquinoline‐3‐carbonitriles on Fe(110), where red regions can be seen between iron and C/O atoms, confirming the formation of Fe–C and Fe–O coordination bonds [40]. These results suggest that cholic acid can readily and effectively bind and functionalize MXene surfaces with strong affinity using a non‐covalent adsorption motif and maintain the intrinsic electronic properties of the underlying MXene. This interaction is advantageous in applications where it is needed to modulate interfacial chemistry, such as surface wettability, biocompatibility, or ion sieving, without adversely affecting the intrinsic physicochemical and immunomodulatory properties of MXenes. A similar trend is anticipated for surface modification of MXenes with different bile acids, due to their close chemical compositions; also, the other MXenes, which need to be validated.

### 3.4 Potential Synergistic Immunomodulatory Role of Gut Microfold (M) Cells on Dendrite Cells

Generally, dendritic cells are specialized immune cells that act as messengers, linking the innate and adaptive immune responses by capturing and presenting foreign antigens to T cells. Indeed, dendrite cells play a dual role in triggering specific immune responses and maintaining tolerance to self‐antigens, as well as their inherent immunosuppressive functions, which involve inducing tolerance to activated T‐cells, promoting regulatory T cells, and inhibiting inflammation. They achieve these modulations by producing inhibitory molecules and altering cytokine profiles, such as interleukin‐10 (IL‐10) and transforming growth factor‐beta (TGF‐β). Smartly, dendritic cells present antigens in a way that triggers tolerance, activating “off‐switches” for the immune system [41]. Accordingly, a recent study reported that human gut M cells can resemble dendritic cells and present gluten antigen, including a detailed mechanism of how these cells inherently contribute to immune modulation and suppression of systemic inflammation toward potential in the gut [42].

**FIGURE 6:**
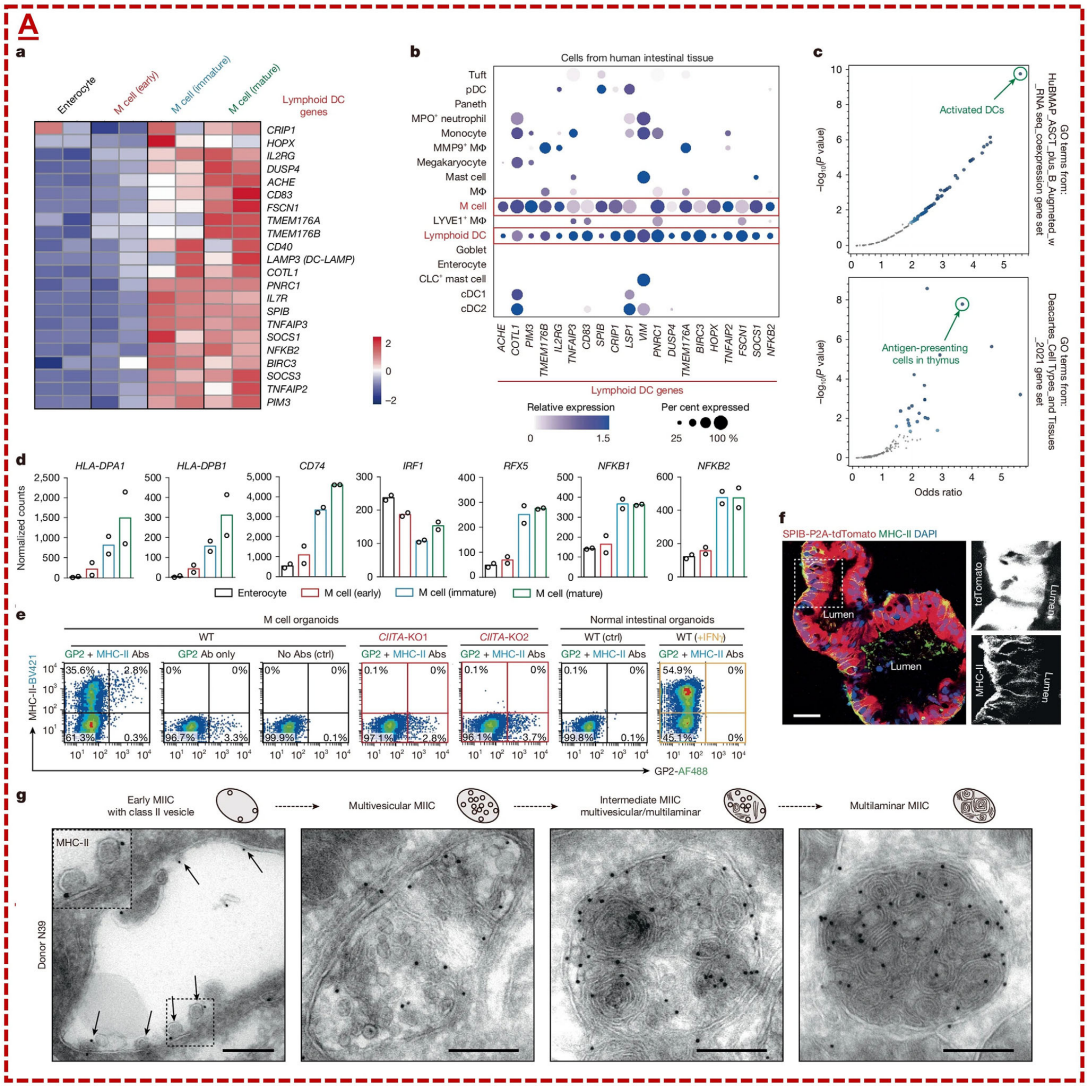
Represented data on the ability of Human M cells to express dendritic cell markers, including the MHC‐II. (A) Heatmap depicting the expression pattern of a lymphoid dendritic cell marker set during the differentiation of M cells (N = 2), as well as a dot plot showing their expression levels of (cluster 9 markers) across multiple immune cell and intestinal epithelial cell types derived from tissue (scRNA‐seq datasets, n = 20, 210 single cells). This panel also represents the volcano plot showing the gene ontology analysis in M cells based on the upregulated differentially expressed genes, identifying the most relevant cell types (activated dendritic cells: top, and antigen‐presenting cells: bottom, *p*‐values are obtained from 1‐sided Fisher's exact test method). It also depicts the normalized expression levels of MHC‐II related genes across these cell populations, as well as representative MHC‐II and GP2 expression cytometry analysis in the cultured M cell organoids (wild type vs. normal intestinal organoids and with/without IFNγ). Multiple independent tests were carried out in wild‐type organoids derived from three different donors and 2 different CIITA‐knockout clonal donor‐derived organoid lines. Representative confocal microscopy imaging and immuno‐electron microscopic illustrations showing MIIC structures in endosomes (in this data, it was identified by immunogold staining of MHC‐II antibody in M cells organoids) with direction of arrows, indicating MHC‐II vesicles in endosome scale bars‐ 200 nm. Adapted with permission from [42], Nature 2025, Open‐access copyright).

Their results suggested that human M cells, which reside in the follicle‐associated epithelium of Peyer's patches, not only contribute to transporting antigens, but also act as robust antigen‐presenting cells with characteristics similar to dendritic cells [42]. Using a rational organoid model, they have achieved to reconstruct the differentiation route of M cells, suggesting that these cells could readily and effectively express various dendritic cell‐related genes, including the class II‐ major histocompatibility complex (MHC‐II) molecules, without being dependent to inflammatory signals, including interferon‐gamma (IFN‐γ), which is responsible for making them capable of intrinsic antigen presentation under homeostatic conditions. Likewise, dendritic cells, the M cells in their study, also require differentiation factors, such as receptor activator of nuclear factor‐κB Ligand (RANKL), colony‐stimulating factor 2 (CSF2), transcription factor Spi‐B (SPIB), and runt‐related transcription factor 2 (RUNX2). Furthermore, they present phagocytic activities and T‐cell interactions, supporting the underlying mechanisms of antigen‐presenting cells (see Figure 6 for the adapted data on the ability of M cells to express dendritic cell markers, including MHC‐II) [42].

In particular, the authors in that work demonstrate that human M cells could take up gluten peptides (gliadin) and present them via “HLA‐DQ2.5”, which is referred to the specific protein heterodimer formed by the HLA‐DQA1^*^05 and HLA‐DQB1^*^02 alleles, particularly the combined protein (DQA105 / DQB102), and is a particular heterodimer variant of the MHC Class‐II molecules. They applied HLA‐DQ2.5 to activate gluten‐specific CD4^+^ T cells, directly involving M cells in coeliac disease pathogenesis. Their organoid T‐cells co‐culture data suggested that MHC‐II and M cells could initiate antigen‐specific T‐cell activation and subsequent proliferation, apart from enterocytes, even when MHC‐II is expressed. Together, their pilot findings suggest that M cells have a key role in shaping mucosal adaptive immune responses, which can potentially influence the outcomes in similar conditions and other gut‐related immune disorders. This progress might indirectly link with the proposed approach in the current perspective on gavage administration of MXene for immunosuppression and systemic inflammation reduction after organ transplantation, and for the treatment of autoimmune diseases beyond the gut sites (details on the ability of M cells to present gluten antigen for T cell activation and immunomodulation are in the original paper).

Even though the referred paper did not specifically report that M cells are immunosuppressive and/or able to indirectly alleviate or control systemic inflammation, beyond direct bloodstream injection, we speculated that the inherent mechanisms of M cell antigen sampling can also influence immune responses both locally and systemically. This hypothesis further supports and highlights the rationale behind the proposed gavage administration of surface‐modified MXenes for immunosuppression with significantly lower risks of longer‐term nanotoxicity and potential tissue accumulation or blood aggregation. Notably, to date, no in vivo studies have reported the complete degradation or robust bio‐clearance route of MXenes after direct intravenous injection into the bloodstream of laboratory animals. Taking these accounts into consideration, it is anticipated that under normal conditions, M cells facilitate immune surveillance by transporting luminal antigens and inducing adaptive immunity and tolerance to foreign/harmless antigens, substantially inducing systemic immunity beyond the intestinal niche. This rational idea may support the multi‐synergetic immunosuppression functions of gavage‐administered MXene nano‐dispersions and their parallel interactions with the gut's beneficial microbiome and systemic immunomodulation. Antigen presentation influenced by M cells within gut‐related lymphoid tissues may induce T cell priming for regulatory signals and inflammation in susceptible individuals.

From biological perspectives, the concept that gut M cells can act as antigen‐presenting cells, providing support for considering oral or gavage delivery of nano‐engineered materials, including MXenes, as an alternative to intravenous administration, is emerging. These cells are specialized to transport macromolecules, particulate compounds, or synthetic nanoscale materials from the intestinal lumen to gut‐associated lymphoid tissue, where immune responses are initiated and/or regulated. This theory potentially holds the capacity to open up opportunities to exploit physiological immune entry routes (e.g., gavage administration) rather than bypassing them via direct bloodstream injection, enabling more concise and controlled immunosuppression along with improved mucosal targeting with reduced risks of systemic toxicity for future nano‐immunotherapies and next‐generation combined strategies.

### 3.5 Standard Dose Considerations, In Vitro/Vivo Validation‐Optimization, and Outlooks

The concentration of starting MXene for gavage treatment, as well as the dosage of cholic acid or any other bile acid surface coatings, needs to be carefully tested and optimized based on currently existing ranges and clinical thresholds. Based on safe dosage guidelines and reasonable ranges for binding and gavage treatment. The starting concentration range should be low enough to minimize the risk of nanotoxicity or excessive bioaccumulation of bile acids, but also sufficient to enable effective working of immunosuppressive nanoparticles and the proposed surface modification. The working concentration of MXenes may be started with a recent publication on immunoengineering applications or drug delivery of these systems. In the optimized dosage range of nanoparticles, sufficient amounts of cholic acid or other potential bile acid coatings can be reasonably studied. It is important to avoid coating the nanoparticles without overwhelming the system. The typical range for bile acid‐based compounds used in pharmacology is recommended.

Furthermore, testing and optimization, both in vitro and in vivo, must be considered. Before proceeding with in vivo experiments, it is rational and ethical to perform sufficient in vitro testing to not only assess and optimize the biocompatibility and nanotoxicity of the nanoparticles, particularly with immune cells and endothelial cells in single‐cell or co‐culture systems, but also to ensure that the concentration of cholic acid or other relevant bile acids does not adversely affect cell viability, excessive ROS production, and/or induce immune activation. If moving forward with laboratory animal studies, the dosage of surface‐modified MXenes or other immunosuppressive nanoparticles must scale up gradually, along with monitoring for any possible adverse effects, such as gastrointestinal irritation or uncontrolled immune responses. When determining effective doses for gavage administration of bile acid‐modified MXene nanoparticles, the body weight of rats or mice must also be considered to avoid exceeding the standard protocols of animal surgeries. According to the literature, biocompatible doses of MXenes vary in different biological systems. However, most of these studies reported that MXene biomaterials, including their nanosheets and quantum dots, possess biocompatibility with various human/mammalian cell types at controlled doses often ≤50 or 100 µg mL^−1^, at least at short‐term timepoints (e.g., ≤24–72 or up to 120 h) [12, 13, 14, 15, 16, 17, 19, 20, 21, 22]. The ProTox‐3.0 models predicted the LD50 toxicity of bile acids to be ≤1000–5000 mg kg^−1^.

Monitoring and adjustments are another critical step in carefully controlling the process and avoiding any complications or issues with tested animals, including all ethical considerations and adjustments based on animals’ responses and experimental conditions. The time course would also be determinant in optimizing the effective dose and number of gavage treatments, as the immune suppression results of orally delivered MXene may vary with direct intravenous injection, especially for autoimmune diseases, for which early‐stage treatment is not urgent, such as transplantation. The time point of intervention for the immunosuppressive effects of gavage administration of MXenes also varies relative to transplant timing. Administering the surface‐modified nanoparticles before the transplant may help in preinducing systemic immune tolerance and overall host immune responses to the donor cells/organs. Gavage treatment, before and after transplantation, may also be considered based on rational experiments and at optimal doses of bile acid‐MXenes. Obviously, the assays must be stopped in case of any irregular in vivo observations.

The feasibility and efficiency of the proposed methods are largely dependent on the quality and purity of MXenes and their coating outputs. Therefore, it is highly recommended to apply only large‐studied or optimized compositions and structures of MXenes. In practice and pre‐clinical studies of this concept, the development of oral immunosuppressive therapies requires multidisciplinary knowledge and collaboration among scientists with diverse backgrounds, with the ultimate aim of a deep understanding of MXenes’ nano‐bio interactions with different immune cells and the gut microenvironments. Further, the synergetic immunomodulatory effects of MXene on T cells, M cells, B cells, and their transferring or mobile signaling from the gut to distinct organs, including transplantation sites, particularly in the context of inflammation or transplant rejection, need to be extensively studied and optimized. Antigen presentation in the gut‐associated lymphoid tissues and the beneficial microbiome are another important aspect of future mechanistic studies.

Moreover, there may be various similarities between the proposed method and existing therapeutic mechanisms through which and how immunosuppressive drugs are currently applied in medical practices. This includes both types of intravenous and oral administration of bioactive particles aimed to suppress the immune response post‐transplantation, controlling autoimmune responses, and treating chronic inflammatory conditions. We briefly speculate on the underlying nanostrategies with actual immunosuppressant therapies in terms of the function of drugs vs. immunomodulatory properties of nanomaterials. It is therefore anticipated that, in addition to the lack of necessity for long‐term consumption of immunosuppression drugs, due to the ability of MXene (and other potential nanomaterials) to target specific tissues and cells, they may cause fewer systemic adverse effects than drugs. The metabolism and related breakdown of the immunosuppressant drugs are another limitation, as most drugs are metabolized in the liver. Orally delivered drugs are chemically modified by associated enzymes (primarily those in the cytochrome P450 enzyme system), followed by being processed through Phase I & II metabolisms for oxidation, reduction, and hydrolysis, as well as biomolecule conjugation (e.g., sugar or sulfate groups). Most water‐soluble drugs and associated metabolites are mainly excreted through the kidneys via urine. Therefore, in addition to drug‐half‐life limitation, weakening of the immune system and the risk of kidney dysfunction are possible over time. If successful, gavage delivery of surface‐modified MXenes holds potential for future immunosuppression therapies. Phagocytosis and other related immunological and biological mechanisms may also play a role to facilitating the breakdown of bile acid‐coated particles into smaller sizes, resulting in a more effective bioclearance. The proposed method may be stopped once the patient is stable and no longer needs it for the rest of their life after transplantation. Nonetheless, these theoretical concepts need to be robustly validated.

In terms of potential risks, it is crucial to mention that although gavage delivery of bile acid‐coated MXenes into the gut is a rational and route‐specific method, the induction of such minor size or form‐dependent physical or biomechanical stress is susceptible. However, this minor localized irritation can be potentially mitigated not only by the anti‐inflammatory effects of MXene but also by the impact of *Bacteroides* species, particularly *B. xylanisolvens* and *B. cellulosilyticus*, which naturally exist in the body and play a key role in suppressing intestinal inflammation. A recent study reported the insights into immunoregulatory ability of *Bacteroides cellulosilyticus and xylanisolvens* human gut microbiota isolates [43, 44]. Their findings conclude that the alterations in the abundance of *Bacteroides* species are directly/indirectly linked to the significant disruption of the intestinal epithelial barrier and chronic inflammation. The authors discussed that these *Bacteroides* isolates could readily and effectively reduce inflammation in *Colorectal Adenocarcinoma 2* cells (Caco‐2) intestinal epithelial cells through decreasing the IL‐8 chemokine levels and transcription of NF‐kB, and the production of proinflammatory TNF‐α and IL‐1β cytokines, as well as inhibiting the Th_1_‐polarizing IFN‐γ cytokine and modulating CD4^+^ T‐cell homeostasis for adaptive immunity. Thus, these *Bacteroides* strains and bile acid‐coated MXene may synergistically contribute to further maintaining the integrity of the intestinal barrier and reducing both local and systemic inflammation by directly modulating the immune cell responses and activating anti‐inflammatory pathways. This microbial modulation may also act as a protective mechanism against the mild physical damage/inflammation that may be induced by gavage administration of nanoparticles; Thus, maintaining the overall gut health and reducing intestinal irritation. This scenario primarily focuses on the immunosuppression role of bile acid‐coated MXene in the context of systemic effects beyond the gut, with the secondary aid of local gut immunomodulation.

### 3.6 Proposed Mechanistic Basis and Consideration of Gavage Bile/MXene Immunosuppression

To outline the hypothesized pathways through which orally delivered MXenes are expected to exert immunosuppressive effects, a set of structured experimental frameworks is essential to evidence the systemic and local gavage‐mediated immunomodulation. First, in vitro assays must assess the stability of optimal bile acid‐coated MXenes under robust simulated gastric and intestinal conditions. These parametric studies encompass bile acid‐modified surface stabilization, pH‐dependent biodegradation of particles, and enzymatic exposure, alongside acceptable biocompatibility with distinct related cells, including the intestinal epithelial cells and dendritic cells, to validate the negligible cytotoxicity of nanoparticles and selective uptake mechanisms. Second, extensive mechanistic in vivo models are required to determine the feasibility and efficiency of orally delivered bile acid‐MXenes in suppressing local and systemic inflammation and preventing donor cells/organ transplant rejection, while monitoring long‐term organ‐specific toxicity, biodistribution, molecular/biochemical signaling, and sufficient bioclearance rates. Third, mechanistic investigations should examine the underlying biological/immunological interactions of bile acid‐coated MXenes with M cells, dendritic cells (and other related immune cells), as well as the gut microbiota. This includes how each bile acid functionalization could modulate microbial composition, secondary metabolism, and subsequently FXR/TGR5‐mediated tolerogenic immunomodulatory mechanisms. Finally, the dose optimization, introducing optimal compositions/forms of bile acid/MXene formulations and their administration regimen studies are essential to design and develop pharmacokinetic models, local mucosal exposure optimization, establishing lymphatic transport efficiency, and tolerogenic immune outcomes/inflammation suppression mechanisms, providing a foundation for further in vivo investigation and pre‐clinical settings with the ultimate goal of clinical translation of this multi‐tiered rationale strategy.

Upon oral administration, the nano‐dispersions encounter a dynamic gastrointestinal environment that influences their surface stability, protein conjugation, enzymatic/cleavage effects, bio‐transports, and enrolment for underlying immunomodulatory activities. In the stomach, and particularly gut areas, the relatively low pH and pepsin induce protonation, lattice disruption, partial oxidation, and colloidal aggregation, rather than the bile salts, pancreatic enzymes, and luminal proteins in the small intestine induce surface remodeling of particles, including the spontaneous formation of a dynamic protein/bile acid corona that can affect their bio‐stability, mucus penetration, and interaction with epithelial cells. If uncoated MXene dispersions are delivered, they likely reach the follicle‐associated epithelium of Peyer's patches, where M cells transcytose the particles to the subepithelial dome, followed by a fraction internalization by intestinal epithelial cells, most probably through the caveolin‐ or clathrin‐mediated endocytosis and macropinocytosis, subsequently processed by subepithelial dendritic cells. These cells can patently promote a semi‐mature, tolerogenic phenotype, secreting TGF‐β and IL‐10, and inducing regulatory T cells for localized mucosal immunosuppression responses. The non‐absorbed nanoparticles are likely excreted fecally. If the proposed partial systemic exposure occurs through the mesenteric lymphatics, it can limitly modulate peripheral immune engagement without the higher risks of systemic nanotoxicity induced by intravenously injected particles.

Bile acid modification is expected to significantly enhance the in situ stability of gavaged MXenes, their intestinal uptake, and immunosuppressive potential by improving colloidal stability, epithelial recognition, and mucus penetration through engagement with bile acid transporters, which can facilitate M cell transcytosis and epithelial endocytosis. Notably, liposomes and polymeric micelles may also have the capacity to protect MXenes from gastric degradation, maintaining their colloidal stability, enabling slower controlled release in the small intestine, and enhancing lymphatic transport to be collectively uptaken by M cells and dendrites for effective immunosuppression. From a translational perspective, gavage administration can provide localized mucosal immunomodulation, reducing the associated risk of systemic toxicity and the potential interaction with beneficial microbiota. Partial lymphatic transport may enable less efficient systemic exposure compared to intravenous injection; however, dose ranges, treatment frequency, and bioclearance pathways can be optimized and enhanced with bile acid coating to more safely sustain tolerogenic effects and minimize nanoparticle injection off‐target risks.

Another possible gut‐mediated immunomodulation mechanism by gavage bile acid‐coated MXenes is through direct interaction and polarization of local macrophages within the intestinal lamina propria. The gavaged particles may be internalized by gut‐area resident macrophages due to their unique physicochemical and surface properties, alongside microbiota‐mediated bile acid metabolites, which can further bias macrophages toward an M2‐like anti‐inflammatory phenotype through promoting the production of IL‐10 and TGF‐β and simultaneously suppressing local proinflammatory TNF‐α or IL‐6. Systemic anti‐inflammatory effects and immunosuppression can also be directed through lymphatic transport pathways. Although gavage delivery primarily targets the gut areas, likely, a portion of bile acid‐coated MXene dispersions can also reach the mesenteric lymphatics and systemic circulation. Through this route, M2‐biased macrophages or tolerogenic dendritic cells existing in lymph nodes can produce systemic IL‐10 or TGF‐β, which can circulate to other tissues before being digested and excreted from the body, while effectively modulating peripheral M1 macrophages. This mechanism can indirectly reduce the production of TNF‐α or IL‐1β/IL‐6, supporting systemic immunosuppression. Partial bile acid/MXene exposure in the spleen or liver resident macrophages, including Kupffer cells, also has the capacity to boost systemic immunosuppression by promoting anti‐inflammatory phenotypes. Another important aspect is that intravenous injection of MXenes can primarily expose circulating immune cells, bypassing gut‐mediated tolerogenic autophagy‐related pathways. Additionally, gavage delivery can engage gut immune hubs, where positive autophagy can be directed and coordinated through interactions with microbiota, bile acid signaling, and FoxP3^+^ Treg induction, enabling more durable physiologically relevant immunosuppression and immune recalibration/programing for inflammation therapies.

Lastly, these features’ capacity can provide a plausible rationale for preferential engagement of mucosal antigen‐sampling cells compared to many conventional lipid‐based, polymeric, or inorganic nanomaterials with enhanced stability in the gut's acidic medium. The proposed gavage application remains to be experimentally validated through robust studies, without presuming superiority of optimal bile acid‐MXene designs over other synthetic immunosuppressive nano‐platforms. Table 2 represents some adapted results of the reported adverse effects of previous generations of carbon‐based nanomaterials on gut microbiota. Notably, several other publications reported the positive effects of carbon nanomaterials on gut beneficial organisms. These dose‐dependent behaviors and material properties underscore the need for comparable bio‐experiments.

**TABLE 2:** A tabulation on some of the adverse interactions of synthetic materials with beneficial gut microbiota and disrupted immune regulation at the tested dose and experimental conditions. These representative findings in the literature highlight the importance of designing biocompatible and surface‐modified materials for gavage delivery of nano‐immunosuppressants. These contents have been adapted from the referenced papers without independent assessment of their accuracy.

Synthetic nanomaterials	Size range	Dosage and Exposure time	Impact on gut microbiome	Effect on inflammation	Immune interactions	Reported outputs	Ref
Monolayer graphene nanosheets	∼200–500 nm	1 µg per day for 21 days	–↓ *Bacteroidetes* abundance −↑ *Lactobacillus*	Decreased intestinal diversity & disrupted immunoregulation	Altered M cells/immune response via microbe shift	Reduced the effectiveness of immune regulation	[45, 46]
Graphene oxide nanosheets	∼100–300 nm	200 mg kg–^1^ per day for 28 days	−↓ *Lactobacillus* −↑ *Firmicutes*, Proteobacteria	‐ Increased ROS production, & inflammation in gut	Impaired immune regulation & disrupted M‐cells activity	Caused gut microbiota dysbiosis & damaged intestinal cells, immune dysfunctions	[45, 47]
Graphene oxide nanosheets	∼200 nm	25 and 250 mg L–^1^ for 2 h	−↓ *Bacteroidota* Adverse shift in the F/B ratio	Changes in gut microbiota composition & inducted inflammation	Adversely affected immune modulation & interaction with M‐cells	Disrupted gut microbiota diversity at tested doses and immune homeostasis.	[45, 48]
Reduced graphene oxide sheets	∼200–500 nm	Dietary exposure at 1 µg per day for 21 days	−↓ *Bacteroidetes* −↑ *Lactobacillus*	Disrupted gut microbiota & decreased overall intestinal health	Impaired immune regulation & M cells‐mediated response	Disrupted microbiota diversity, impaired immune function and gut barrier damage & inflammation	[45, 46]
Graphene oxide nanosheets	≥500 nm	120 mg kg–^1^ per every 3 days for tested 16 days	Adverse shift in gut microbiota composition: Increased Proteobacteria, decreased Firmicutes	Induced intestinal damage & with risk of systemic inflammation	Affected M‐cells & decreased reduced immune function	Induced intestinal damage and immune dysfunction, impaired gut immunotolerance & inflammation	[45, 49]
Pristine graphene (flakes/sheets)	∼1–2 µm	1, 10, 100 µg per day for 4 weeks	−↓ *Lactobacillus* −↑ *Prevotella*, *Anaeroplasma*	Induced liver damage through increased ROS with inflammation risk	Disrupted M cells & immune cells function	Altered microbiota composition, increased ROS, liver damage, impaired immune process/gut health	[45, 50]
Silver nanoparticles	∼10–100 nm	1, 10, 100 µg mL–^1^ for 3, 6, and 24 h	Disrupted useful bacteria *L. casei*, *L. plantarum*, *L. fermentum* Adverse ratio shifts: *Firmicute*	Increased proinflammatory cytokines & disrupted microbial homeostasis	Adversely impacted on probiotics (*Bacillus subtilis*) & disruption	Antimicrobial effects disrupt gut microbial balance & Increased inflammation	[51, 52]
Titanium dioxide nanoparticles	∼10–100 nm	25 and 250 mg L–^1^ for ∼72 h	Depleted *Lactobacillus* & Disrupted gut microbiota composition	Adversely Induced colonic inflammation through NF‐κB activation	Adversely effected *Lactobacillus rhamnosus* Grigory G	Induced microbiota dysbiosis & inflammation need probiotics for protection	[51, 53, 54]

### 3.7 Potential off‐Target Effects of Gavaging Bile Acid‐MXenes and Translational Frameworks for Balancing Functionality, Systemic Efficacy, and Safety

While gavage delivery may induce lower exposure kinetics and hemocompatibility, as well as endothelial stress, compared to intravenous injection, potential off‐target effects need to be critically considered. This includes explicit statements and robust studies to clarify the method's outcomes and validate that the significant effects of MXene composition/quality/purity, bile acid type, and effective coating, and in situ oxidation stability of optimal bile acid‐modified MXenes in gastric and intestinal environments, as well as the particle aggregation state, and working dosages. In addition, their interaction with M cells and dendritic cells must be considered and optimized as a property‐driven view rather than a cell‐exclusive frame, arising from the interplay of material properties with the gut and intestinal microenvironments.

These off‐target effects must be given careful consideration in light of the central role of bile acids in gut homeostasis and underlying systemic metabolism mechanisms. Indeed, bile acid modification may influence gut microbiota composition, as specific commensal bacteria metabolize bile acid coatings and are sensitive to charged surfaces. Thus, if bile acid/MXene designs have not been appropriately engineered or have been delivered with non‐optimized doses, it may be relatively disruptive to the beneficial bacteria in the gut, alter short‐chain fatty acid production, impair barrier function, or even result in negative shifts in immune responses. Furthermore, if MXenes are overloaded with high bile acid dosages, they may adversely impact host bile acid receptors, including the farnesoid X receptor (FXR) and the G‐protein‐coupled bile acid receptor (TGR5), expressed in enteroendocrine cells, intestinal epithelial cells, and immune cells. The overstimulation of these receptors may also result in alterations in glucose and hepatic lipid metabolism, secretion of gut peptides such as glucagon‐like peptide 1 (GLP‐1) and peptide YY (PYY), and mucosal immune signaling, imposing unwanted metabolic or immune activations. Therefore, to validate the feasibility of rationally proposed hypothesis‐driven gavage methods, MXene composition/form, surface chemistry, bile acid coating parameters, as well as the mucosal uptake and interaction with antigen‐presenting cells and beneficial gut microbiota, require empirical research investigation. These bioassays include microbiome profiling, receptor activation evaluation, and metabolomic analysis. Indeed, by incorporating this framework, the immunosuppressive benefits of bile acid‐functionalized MXenes and their controlled microbial and metabolic modulations can be robustly validated, providing a realistic trade‐off perspective on their real‐world translation in future therapeutic applications beyond intravenous administration and its currently existing associated safety/efficacy limitations.

### 3.8 Proposed In Vivo Transport Mechanism of MXene after Gavage Administration

It is well known that orally administered engineered nanomaterials have the potential for antimicrobial applications and uses in food packaging. Orally delivered nano‐substances can be cleared at certain amounts through the mucociliary escalator cells into the cavity and subsequently into the intestinal tract [55]. Even the synthetic nanomaterials deposited in the skin may subsequently reach the gut lumen. On this basis, it is assumed that the human intestinal tract is partly and often involved in the biological processing of synthetic nanomaterials sourced from various exposure routes, including food additives, packaging, agricultural foods, contaminated water, dietary supplements, and hand‐to‐mouth transfer. While orally‐administered non‐bioactive nanoparticles mostly stay in the gut and are biologically eliminated, small amounts of them may cross the intestinal barrier, interact with gut microbiota, and even enter the lymphatic system.

As part of the natural body's immune/defense responses against invading foreign agents, the intestinal tract is one of the primary target sites. If the given nanoparticles possess high biocompatibility or inertness with the gut area and microbiota, the toxicity reaction may be negligible; However, high doses or non‐biocompatible particles can induce toxicity to the intestinal tract and adversely affect the gut beneficial bacteria. This concern may also be valid for MXene, as investigation on the effect on intestinal components, including epithelial cells, gut microbiota, mucus layer, and the gut immune system, is in its early stages. This highlights the rationale behind efficnet bile acid coating, recognized as a “self” or, at least, not being identified as invading agents or pathogens.

While the exact transport route and undelaying transport bio‐mechanisms of bile acid‐MXene particles are unknown and need to be experimentally evidenced, physiologically related pathways can be proposed based on other materials, particularly carbon‐based nanostructures. This intestinal immunophysiology includes the likelihood of intestinal uptake, M‐cell‐mediated transcytosis, and enterocyte endocytosis, epithelial transport, and Peyer's patch‐specific mechanisms. Following gavage administration, the given particles with enhanced physicochemical/biological properties may first encounter the intestinal mucus layer, which acts as a charge‐ and size‐selective diffusion barrier. Protein corona conjugated nanoparticles may penetrate the mucus layer and interact with the intestinal epithelium. The resultant translocation across the epithelial barrier is attributed to transcellular transport and paracellular transport routes. Transcellular uptake is mediated by endocytic mechanisms in enterocytes (i.e., clathrin‐mediated endocytosis, caveolae‐dependent endocytosis, and micropinocytosis). In addition, M‐cells located within the follicle‐associated epithelium of Peyer's patches may play a pivotal role in particulate antigen sampling, facilitating the uptake and transcytosis of particles from the intestinal lumen to underlying immune cells. Their delivery into gut‐associated lymphoid tissue may subsequently lead to entry into lymphatic circulation. For metallic atoms of bile acid‐coated MXenes, the transcellular and M‐cell‐associated pathways are likely mechanisms of systemic entry. Upon epithelial translocation, the given particles may indirectly access the systemic micro‐circulation, likely through mesenteric lymphatic drainage or the portal venous system [45, 56].

Notably, the purity, size, surface chemistry, charge, efficient bile acid attachment, and compatible interaction with immune/endothelial cells are determinative factors that can influence macrophage uptake and positive interaction with dendritic cells, which can further lead to influencing proinflammatory cytokine production and subsequently immune suppression signaling. Indeed, gavage administration of bile acid‐modified MXenes may offer benefits, including their capacity to be partially degraded and broken down into carbon‐based substances by gut microbiota as a carbon source for metabolism. The microbes metabolize these compounds into smaller fragments and convert them into useful metabolites, such as butyrate, which is a short‐chain fatty acid. Additionally, optimal bile acid‐coated MXenes may contribute to promoting the balance of beneficial microbes under stress by modulating ROS, which, with their immune suppressive and antipathogenic activities, facilitate their systemic immunomodulation and bioclearance. Lastly, as the delivery induces lower or delayed systemic exposures compared to direct intravenous injection due to incomplete absorption, first‐pass hepatic processing, or epithelial barrier constraints, a reduced peak plasma concentration can also be obtained.

### 3.9 Proposed In Vivo Impact of Transplant Types (Allograft, Heart, Kidney, Liver, Retinal) on the Gavage Administration Regimen

From an immunological perspective, the immune rejection pathways significantly differ among the types of donor transplants and medical patient conditions. In particular, allograft organs, due to their variations in vascularization, local immune regulation, and antigen presentation dynamics, act differently from vascularized solid organs, such as the heart and kidney, whose rejection is predominantly driven by direct/indirect allorecognition pathways. Post‐transplantation of complex organs triggers the host immune responses, activating CD4^+^ and CD8^+^ T‐cells, as well as donor‐specific antibody production, which, along with endothelial injury, may further result in acute and chronic rejection with essential immunosuppressant therapies. In the case of liver transplants, the occurrence of partial tolerogenicity is possible, which can subsequently be attributed to hepatic antigen‐presenting cells, such as Kupffer cells and liver sinusoidal endothelial cells, and T‐cell induction. However, alloimmune response may still result in fibrosis and graft dysfunction, leading to organ rejection. In the case of retinal transplants and hepatic allografts, which include a relative immune privilege or intrinsic tolerogenic microenvironment, they can be influenced by the blood‐retinal barrier, microglial activation, and/or low baseline MHC expression, even though the inflammatory conditions may also occur upon barrier disruption. The regulatory antigen‐presenting cells, local IL‐10 and TGF‐β production, and reduced costimulatory signaling are expected to require less intense systemic suppression to prevent rejection. In donor cell‐based transplants, including stem cells and hematopoietic cell transplantations, innate immune recognition and systemic macrophage clearance/damage interact with adaptive alloimmune mechanisms, increasing the risk of cell survival and their rejection. Given these mechanistic differences across distinct transplants, the anticipated immunosuppressive effects and underlying mechanisms of gavage‐administered MXenes benefit from the intrinsic immunomodulatory behavior alongside potential mediation of gut‐associated lymphoid tissue for enhanced regulatory mechanisms of T‐cells and systemic cytokine modulation.

Nevertheless, the potential immunologic impacts of bile acid‐MXenes on distinct transplant types when administered by gavage may be common in interacting with gut‐associated lymphoid tissue, intestinal dendritic cells, Peyer's patches, and the gut microbiome, as well as systemic immunosuppression through promoting the regulatory T cells (i.e., FoxP3^+^), expanding tolerogenic dendritic cells differentiation, and suppressing Th_1_/Th_17_ cytokine axes (e.g., IL‐17, TNF‐α, IFN‐γ). The anticipated mechanisms may potentially favor immune reprogramming rather than global lymphocyte suppression. Such gut‐mediated tolerance induction may be slightly different in liver or hematopoietic stem cell transplantations, where modulation of intestinal immune activation can reduce graft‐vs.‐host responses. Moderate benefit may also occur in kidney transplants, where indirect inflammatory cytokine signaling and allorecognition mainly contribute to organ rejection. Using the gut to induce/create immune tolerance by oral gavage of bile acid‐MXene may also lead to expression of CCR7 and a4β7 to educate the immune cells/system and effectively suppress inflammation after transplantation. However, in highly immunogenic, vascularized transplants, such as the heart, gavage strategies may need to be hybridized with a limited frequency dose of immunosuppressant drugs, especially at the early stages of transplantation, to suppress inflammation and antibody‐mediated responses. Using enteric capsules that can pass through the stomach and release their contents only in the higher pH environment of the small intestine, and the intraperitoneal administration of bile acid‐modified MXene may more effectively deliver the bile acid‐modified MXene particles directly to the peritoneal cavity and abdominal organs (e.g., liver and intestines). This hybrid method may help absorb immunosuppressive particles into the bloodstream, albeit with a lower distribution capacity/rate, compared to intravenous injection. Thus, these speculations on the comparative evaluation across different transplant models are essential to be experimentally validated and optimized context‐dependently and parametrically.

## 4 Conclusion and Future Outlook

To conclude, this opinion‐based perspective aims at two primary objectives. First, we aim to highlight the recently obtained research findings on the intrinsic immunosuppressive properties of specific MXenes and their anti‐inflammatory roles in preventing transplant rejection and treating inflammatory diseases. Second, we aimed to propose an innovative route‐specific alternative to intravenous injection into the bloodstream of research animals, which has been the most common administration method, as well as for the delivery of immunosuppressant drugs at early stages post‐transplantation. The ultimate goal is designing safer and more efficient in vivo strategies to deliver bile acid‐modified MXenes or other immunosuppressive nano‐dispersions through gavage and direct transfer into the stomach/gut, reducing the associated risks of longer‐term blood nano‐toxicity or particle tissue‐accumulation in circulatory organs such as the heart, liver, kidney, or lungs, as well as potential adverse effects on metabolism or other biological pathways. This alternative may alleviate hematotoxicity concerns while retaining their immunosuppressive roles.

To increase the feasibility of the proposed gavage method, we also present novel surface modification strategies based on bile acid coating that can potentially enhance the MXene‐mediated inflammation‐suppression and anti‐rejection functions, and induce positive interactions with gut‐beneficial bacteria, bringing this nanobiotechnology closer to practical settings. Optimal conjugation with bile acids has several advantages, including the enhancement of colloidal stability of nano‐dispersions likely through micelle‐like interactions, and promoting mucus penetration and recognition/transport through bile acid‐responsive epithelial pathways, which potentially increase transcytosis through endocytosis and M cells by enterocytes. Bile acid‐modified MXenes also positively impact the gut microbiota, promoting the growth of bile‐resistant commensals and enhancing mucosal immune tolerance. To optimize delivery, design strategies and hybrid medication approaches are recommended for research studies to validate their efficient immunosuppression and reproducibility over different biosystems. From translational outlooks, gavage administration may offer advantages over intravenous delivery due to its potential to reduce systemic nanotoxicity and enhance tolerogenic immune responses. An experimental roadmap can include systemic simulated gastric and intestinal fluid stability bioassays, gut microbiota/organoid co‐culture systems, biodistribution and bioclearance analysis, and comparative toxicity assessment of MXenes with and without bile acid, experimentally and using machine learning and artificial intelligence methods. The ultimate goal is to reduce the need for outpatient intravenous injections.

Some of the future outlooks recommended for experimental and modeling research include:

i In vitro validation of the intestinal stability and cell biocompatibility of bile acid‐MXenes.

In particular, evaluating their stability under physiologically relevant gastrointestinal conditions, including different pH ranges, interaction with mucus, digestive enzymes, and bile salts, bio‐tracking characterization of the administrated particles post‐digestion with respect to charge, aggregation state, structural integrity, protein corona formation, and biodegradation. Furthermore, investigating the interactions between the epithelial barrier and optimal bile acid‐MXene conditions using in vitro intestinal co‐culture, organoid models, or gut‐on‐chip systems to measure permeability. Assessment of biocompatible doses, potential cytotoxicity or oxidative stress production effects, at different time points, as well as optimizing cellular uptake mechanisms, intracellular trafficking, and translocation.

ii In vivo assays to evaluate the inhibitory effect of gavage administration on transplant rejection and long‐term toxicity effects of particles.

In particular, in vivo validation of gavaging optimal bile acid‐MXenes to evaluate their efficacy in suppressing inflammation, attenuating donor transplant rejection, route‐specific biodistribution analyses, organ‐specific histopathology, and immunogenicity safety using well‐established models.

iii Investigating the interaction mechanism between MXene and intestinal flora and M cells.

In particular, detailed and robust investigations of the mechanistic interactions between delivered particles and the intestinal microbiota, as well as optimizing M‐cell‐mediated uptake mechanisms and translocation pathways within the gut‐associated lymphoid tissue and associated immune cells.

iv Optimizing dosage and administration regimen of optimal bile acid‐MXenes to provide a basis for pre‐clinical applications and future translations.

In particular, optimizing dose/treatment frequency parameters under optimal conditions to validate the efficacy of the proposed method paves the way to establishing pharmacokinetic profiles, reproducibility, and safety to support preclinical development and translational progression.

Lastly, several other critical considerations are recommended as a roadmap for new experiments, including nanomaterial designs, biological and biochemical interactions of bile acid‐coated MXenes on ROS modulation, protein/metabolite regulation, gut physiology/paracellular transport cross‐talk, epigenetic analysis, gene programming in different immune cells, sustained immune suppression, and translational combination with immunomodulatory drugs. The safe dosages of bile acid, particularly cholic acid, depend on the context of targeted applications and whether the loaded cholic acid‐MXenes will be considered for pharmaceutical or biomedical applications. Cholic acid has a hydrophobic steroid backbone structure enriched with hydrophilic active hydroxyl groups, making it desirable for particle assembly and facile conjugation methods.

## Author Contributions

This work was conceptualized, designed, and reviewed by A.R. and A.A. A.R., Ak.R., and A.A. drafted the manuscript. A.A. and Ak.R. performed the DFT and MD calculations. All authors approved the manuscript for submission and publication.

## Conflicts of Interest

The authors declare no competing interests or financial disclosures related to this manuscript.
